# Early-Warning Margins for Preamble-Directed Jamming in LoRa: A Dechirp-Domain Sensitivity Framework with Severity-Graded Composite-Channel Stress Testing

**DOI:** 10.3390/s26196123

**Published:** 2026-09-27

**Authors:** Carlos Herrera-Loera, Carolina Del-Valle-Soto, Leonardo J. Valdivia Parga, Carlos Mex-Perera

**Affiliations:** 1 Facultad de Ingeniería, Universidad Panamericana, Álvaro del Portillo 49, Zapopan 45010, Mexico; aherreral@up.edu.mx (C.H.-L.); lvaldivia@up.edu.mx (L.J.V.P.); 2 College of Engineering Technology, Rochester Institute of Technology, Rochester, NY 14623, USA; jmpiee@rit.edu

**Keywords:** LoRa, LPWAN, physical-layer security, jamming detection, one-class anomaly detection, chirp spread spectrum, detection theory, receiver operating characteristic, synchronization

## Abstract

Physical-layer jamming directed at the LoRa preamble may become detectable before substantial degradation of the acquisition structure is evident at the receiver. The practically relevant question is therefore not only whether an attack can eventually be detected, but how much jammer-power headroom a monitor provides before the selected preamble-degradation onset is reached. This paper introduces a detection-theoretic evaluation framework that expresses this pre-degradation warning capability in decibels. Three quantities are defined: the detection sensitivity floor, i.e., the smallest relative jammer level yielding a detection probability of 0.9 at a false-alarm probability of $10^{-2}$; the preamble-based link-degradation onset, i.e., the level at which the preamble symbol error rate increases by five percentage points relative to its jamming-free baseline; and the resulting early-warning margin separating the two thresholds. The resulting early-warning margin is a power-domain measure of jammer-power headroom and should not be interpreted as elapsed warning time. The framework is evaluated over 13 isolated impairment configurations, corresponding to 221 jammer-level operating points, and complemented by a three-step severity-graded composite-channel ladder that jointly combines noise, multipath, co-channel interference, and synchronization impairments, for more than $5\times 10^{5}$ packet-level evaluations. Alongside a convolutional autoencoder and a one-class support vector machine using the same three-channel in-phase, quadrature, and magnitude preamble representation, we evaluate a training-light dechirp-domain peak-to-mean ratio statistic and a simple score-level fusion rule; under independent threshold calibration, the fusion rule preserves the separability of its constituents but, under fading, interference, and synchronization impairments, does not recover the fixed-operating-point sensitivity of the dechirp statistic. Under strong co-channel interference, the proposed scalar statistic reaches the target detection probability at −8.5 dB, whereas the autoencoder does not reach the target detection probability anywhere within the evaluated sweep, corresponding to a sensitivity advantage of more than 14 dB and showing that the observed loss of autoencoder sensitivity is detector-dependent rather than caused by an absence of discriminative information in the received preamble. No detector dominates across all isolated impairments: the dechirp statistic is particularly effective under co-channel interference and synchronization offsets, whereas the autoencoder remains advantageous under severe multipath fading. Some learned-detector margins become negative under moderate additive noise and under severe multipath fading, and the composite-channel ladder shows a progressive degradation of detection sensitivity as joint impairment severity increases. These results provide a detector-agnostic, physically interpretable yardstick for evaluating the pre-degradation sensitivity of LoRa physical-layer jamming monitors.

## 1. Introduction

Low-power wide-area networks built on LoRa now support a broad range of Internet-of-Things applications, from utility metering to agricultural and industrial telemetry [1,2]. Their long communication range relies on chirp spread spectrum (CSS) modulation, which provides substantial processing gain and enables reliable reception at low signal-to-noise ratios. At the same time, the deterministic and publicly specified structure of the LoRa waveform creates a relevant physical-layer attack surface. Because every LoRa frame begins with a known sequence of upchirps, an adversary can target the preamble and disrupt packet acquisition without requiring access to the transmitted payload or successful demodulation of the victim transmission [3,4].

The security literature has addressed this problem along two largely separate tracks. One quantifies the communication damage caused by interference or jamming, reporting bit, symbol, or packet error rates as functions of jammer power, propagation conditions, and attack timing [5,6,7]. The other develops detection mechanisms, including data-driven one-class models trained from jamming-free observations and commonly evaluated through recall, detection probability, or the area under the receiver operating characteristic (ROC) curve [8,9]. Both perspectives are necessary, but neither directly quantifies the separation between the point at which an attack becomes reliably detectable and the point at which the protected communication structure begins to degrade. From an operational perspective, this distinction is important: a detector is most valuable when it reaches its operating point at jammer levels below those that produce an observable deterioration of the LoRa preamble.

This paper makes that separation the primary object of study. We define a power-domain early-warning margin, rather than an elapsed-time warning interval, so that the metric expresses the relative jammer-power headroom available between reliable detection and the selected preamble-degradation onset. Three scalar quantities, all expressed on the same relative jammer-level axis and estimable from a single power sweep, are considered:

- The **detection sensitivity floor** $L_{0.9}^{★}$, defined as the smallest relative jammer level at which a detector achieves $P_{D}=0.9$ at a fixed $P_{\mathrm{FA}}=10^{-2}$;
- The **preamble-based link-degradation onset** $L_{0.05}^{\mathrm{SER}}$, defined as the smallest relative jammer level at which the preamble symbol error rate increases by five percentage points above its jamming-free baseline;
- The **early-warning margin** $M_{0.9}=L_{0.05}^{\mathrm{SER}}-L_{0.9}^{★}$, defined as the jammer-power interval over which the attack satisfies the selected detection criterion before the preamble reaches the selected degradation criterion.

Recasting detector evaluation in these terms has two immediate consequences. First, it makes heterogeneous detectors commensurable: a deep reconstruction model, a kernel one-class classifier, and a closed-form statistic can all be compared through the jammer level required to attain the same $\left(P_{D},P_{\mathrm{FA}}\right)$ operating point, rather than through unitless recall values obtained at potentially different thresholds. Second, it exposes operational failure modes that aggregate metrics can conceal. A detector may exhibit substantial overall class separability while still producing a negative early-warning margin if its required detection level lies beyond the preamble-degradation onset.

To complement the learned detectors with a physically interpretable reference, we introduce a third detector: the **dechirp-domain peak-to-mean ratio** (PMR) of the LoRa preamble. The statistic is obtained by dechirping each preamble symbol against an ideal reference, computing its discrete Fourier transform, and normalizing the dominant-bin magnitude by the mean spectral magnitude. The resulting values are averaged over the eight observed upchirps. PMR therefore measures how strongly the received preamble energy remains concentrated after dechirping and requires no model training, only estimation of its nominal operating threshold. Its computation is also compatible with the dechirp-and-transform operations already used during LoRa acquisition, providing a low-complexity and physically interpretable counterpart to the convolutional autoencoder (CAE) and the one-class support vector machine (OC-SVM).

The comparison enabled by this statistic reveals the paper’s central result. Under strong co-channel interference, the CAE exhibits a pronounced loss of sensitivity, whereas PMR continues to separate jamming-free and jammed observations at substantially lower jammer levels. At a co-channel interference level of 0dB, the CAE does not reach the target detection probability anywhere within the evaluated sweep, whereas PMR reaches the criterion at $L_{0.9}^{★}=-8.45\;\mathrm{dB}$, with an area under the ROC curve of 0.985 at the weakest tested jammer level. Thus, PMR provides a detection-sensitivity advantage of more than 14dB relative to the CAE under this condition. This result shows that the poor CAE sensitivity observed under strong co-channel interference is detector-dependent and does not imply that the received preamble lacks discriminative information about the attack.

Three further findings follow. First, no detector dominates across all isolated impairments. PMR is particularly effective under co-channel interference and synchronization offsets, whereas the CAE provides the strongest sensitivity under severe Rayleigh fading, where the nominal PMR distribution becomes sufficiently dispersed to weaken a lower-tail concentration test. Second, early-warning margins can become negative. Under additive white Gaussian noise at SNR=0dB, the CAE and OC-SVM sensitivity floors lie −0.90dB and −3.28dB, respectively, beyond the preamble-degradation onset, while PMR retains a positive warning margin. Third, jointly impaired channels exhibit behavior that cannot be inferred directly from isolated impairment experiments. Across the three-step severity-graded composite-channel ladder, increasing impairment severity produces a monotonic loss of detection sensitivity: the mean OC-SVM sensitivity floor shifts from −2.33dB in C1 to −1.76dB in C2 and is not attained in C3, while the CAE shifts from +2.59dB to +2.66dB and likewise fails to attain $P_{D}=0.9$ within the evaluated jammer range in C3. In the adverse C3 condition, neither detector attains the target detection probability in any of the four independent runs. The corresponding detector ordering also differs from that observed under some isolated impairments, showing that isolated-condition conclusions do not necessarily transfer to jointly impaired channels.

The contributions of this work are as follows:

1. A decision-theoretic evaluation framework for physical-layer jamming monitors that jointly characterizes the detection sensitivity floor, the preamble-based link-degradation onset, and the resulting early-warning margin on a common relative jammer-level axis (Section 3).
2. A closed-form, training-light dechirp-domain PMR statistic with a one-class test, together with evidence that it provides more than 14dB of additional detection sensitivity relative to the CAE under strong co-channel interference, thereby providing a physically interpretable reference for identifying detector-dependent sensitivity losses (Section 4 and Section 6).
3. A three-step severity-graded composite-channel ladder that shows a monotonic deterioration of detection sensitivity as joint impairment severity increases, and demonstrates that detector rankings obtained under isolated impairments do not necessarily transfer to composite operating conditions (Section 5 and Section 6.6).
4. A quantitative analysis of the statistical complementarity between reconstruction-based and spectral-concentration-based evidence, together with an evaluation of a weight-free score-level fusion rule under independent threshold calibration. The analysis shows that the fusion rule preserves threshold-independent separability but does not necessarily inherit the best constituent sensitivity at the prescribed operating point; across the fading, co-channel, and synchronization conditions analyzed in Section 6.5, its sensitivity floor follows that of the CAE because the standardized dechirp statistic saturates below the fused threshold set by the heavier-tailed reconstruction-error score.

## 2. Related Work and Positioning

### 2.1. Jamming of LoRa Physical Layers

The vulnerability of LoRa and LoRaWAN to intentional interference has been demonstrated through practical attacks, performance evaluations, and dedicated physical-layer studies. Aras et al. [3] showed that selective jamming triggered by preamble detection can disrupt LoRaWAN transmissions using commodity hardware, demonstrating that an adversary does not need to continuously occupy the channel to significantly affect network availability. Perković and Siriščević [4] subsequently demonstrated a low-cost LoRaWAN jammer, reinforcing the practical feasibility of such attacks with readily accessible equipment. Kalokidou et al. [5] further evaluated LoRaWAN resilience under jamming, characterizing the resulting degradation under different jammer operating conditions.

At the physical layer, Hou et al. [10] specifically investigated jamming of the LoRa PHY and corresponding countermeasures, moving the discussion from network-level disruption toward the behavior of intentional interference at the waveform level. More recently, Haque and Saifullah [11] addressed mechanisms for handling jamming attacks in LoRa networks, extending the literature from attack characterization toward mitigation and communication recovery.

The broader security implications of these attacks have also been examined. Hessel et al. [12] place jamming within the wider landscape of LoRaWAN vulnerabilities and mitigation strategies, while the classical study of Xu et al. [13] established the feasibility of both launching and detecting wireless jamming attacks well before the emergence of LoRaWAN. Grover et al. [14] subsequently reviewed the broader family of jamming and anti-jamming techniques in wireless networks, highlighting the dependence of attack effectiveness and defense mechanisms on waveform, timing, power, and receiver behavior.

Taken together, these studies establish that jamming is a practical availability threat to LoRa systems and that the packet-acquisition stage provides a particularly attractive attack surface. However, most existing studies primarily characterize whether an attack disrupts communication, how severely it does so, or how it can be mitigated. The question addressed here is different: at what jammer level does attack evidence become reliably detectable, and how far is that point from measurable degradation of the LoRa preamble?

### 2.2. Jamming Detection and Pre-Degradation Monitoring

Beyond attack characterization and mitigation, several studies have moved toward detecting jamming while useful communication-layer or physical-layer observations remain available. Ruotsalainen [15] proposed reactive-jamming detection for LoRaWAN based on metadata differencing, providing a lightweight alternative to approaches that rely exclusively on physical-layer signal processing. At the physical layer, Alhazbi et al. [16] introduced BloodHound, explicitly targeting early detection and identification of jamming from PHY-layer observations. Sciancalepore et al. [17] subsequently extended this direction through deep learning-based jamming detection in low-BER mobile indoor scenarios, showing that jamming detection can be studied under operating conditions in which communication degradation remains limited.

These studies establish the practical relevance of detecting jamming before complete communication loss. They do not, however, express this advantage as a common physical margin between detector sensitivity and communication degradation. In particular, they do not quantify the separation in relative jammer power between reliable detection at a prescribed $\left(P_{D},P_{\mathrm{FA}}\right)$ operating point and the onset of measurable preamble degradation. This distinction is central to the present work: early detection establishes that an attack can be recognized before complete failure, whereas the early-warning margin quantifies how much jammer-power separation exists between reliable detection and the selected preamble-degradation onset.

### 2.3. LoRa Acquisition, Synchronization, and Signal Processing

The relevance of the preamble as a monitoring region follows directly from the structure of the LoRa physical layer. LoRa relies on chirp spread spectrum modulation, whose basic signal structure and processing principles are described in the Semtech modulation specification [18]. During reception, the deterministic chirp structure of the preamble supports packet detection, synchronization, and subsequent demodulation. This makes the preamble simultaneously essential for reliable acquisition and attractive for physical-layer monitoring.

Software and analytical implementations of LoRa receivers provide additional insight into this processing chain. Robyns et al. [19] developed a multi-channel software decoder for LoRa, illustrating how dechirping and frequency-domain processing can be used to recover LoRa symbols. Marquet et al. [20] further investigated theoretical LoRa performance and demodulation techniques, reinforcing the central role of dechirp-domain processing in symbol recovery. Tapparel and Burg [21] later presented a complete GNU Radio implementation of the LoRa physical layer, providing an open signal-processing framework closely aligned with the waveform generation employed in the present study.

Synchronization has received particular attention because timing and carrier-frequency errors directly modify the signal structure observed during acquisition. Xhonneux et al. [22] proposed a low-complexity synchronization algorithm designed to remain robust to sampling-time offsets. Tapparel et al. [23] specifically addressed LoRa preamble detection in the presence of inter-channel interference, while Tapparel and Burg [24] investigated fine synchronization through joint time- and frequency-offset estimation.

These studies establish two points that are central to the present work. First, the preamble contains deterministic signal structure that can be examined independently of the payload. Second, dechirping maps that structure into a frequency-domain representation in which spectral concentration becomes directly observable. This motivates the use of the first eight preamble upchirps as the monitoring window and provides the physical basis for the dechirp-domain statistic introduced in Section 4.

### 2.4. Impairment Modeling and Error-Rate Analysis

The effects of noise, fading, interference, and propagation conditions on LoRa communication performance have been extensively studied. Afisiadis et al. [6] analyzed the error rate of LoRa modulation in the presence of interference, establishing how competing transmissions alter symbol-level performance. Ferreira Dias et al. [25] derived closed-form bit-error-rate expressions for LoRa under Nakagami and Rice fading channels, while Elshabrawy and Robert [26] developed compact approximations for LoRa bit-error performance.

Structured interference introduces additional complications. Croce et al. [27] demonstrated that the practical orthogonality between LoRa spreading factors is imperfect, so concurrent LoRa transmissions can produce measurable link-level degradation. Georgiou and Raza [28] examined the scalability of LoRa networks and the role of capture effects under concurrent transmissions. Demeslay et al. [7] extended LoRa performance analysis to multipath and interference channels, showing that propagation and structured interference jointly influence receiver performance. At a broader level, Azevedo and Mendonça [29] reviewed the propagation models commonly employed in LoRa studies and the assumptions underlying their use.

This literature provides a mature understanding of how individual physical-layer impairments affect communication performance. Noise modifies the effective signal-to-noise ratio, fading introduces amplitude and phase variability, multipath alters the received chirp structure, co-channel LoRa transmissions introduce structured interference, and timing or carrier-frequency offsets affect acquisition and synchronization.

These works establish how impairments degrade communication. They are silent on the corresponding monitoring problem: how those same impairments reshape the statistical evidence of an attack, and how much detection margin remains before the selected preamble-degradation onset is reached. This distinction is particularly important for one-class detectors because the impairments affect not only the attacked signal but also the jamming-free distribution from which the operating threshold is established. A channel condition may therefore have a limited effect on communication while substantially changing detection sensitivity, or vice versa.

The present study addresses this gap by evaluating detection and preamble degradation on the same relative jammer-level axis. Isolated impairment conditions are first used diagnostically to reveal the failure modes associated with AWGN, severe fading, co-channel interference, and synchronization impairments. These observations are then contrasted with a severity-graded composite-channel ladder in which the impairments act jointly, allowing the transferability of conclusions obtained from isolated conditions to be examined directly.

### 2.5. One-Class Detection and Operating-Point Evaluation

When attack examples are unavailable or cannot be exhaustively represented during training, anomaly detection can naturally be formulated as a one-class problem. The one-class support vector machine introduced by Schölkopf et al. [30], building on the support-vector formulation of Cortes and Vapnik [31], estimates the support of the nominal data distribution and identifies observations that depart from it. This principle is well suited to physical-layer monitoring because large numbers of jamming-free signals can be collected or generated, whereas the waveform, power, timing, and strategy of a future jammer may remain unknown.

Reconstruction-based anomaly detectors provide a complementary approach. Rather than directly estimating the support of the nominal distribution, an autoencoder learns to reproduce nominal observations and uses reconstruction error as an anomaly score. Chalapathy and Chawla [9] survey deep learning approaches to anomaly detection, while Ruff et al. [8] provide a broader unified treatment of shallow and deep anomaly-detection methods and emphasize that different detector families may fail in different regions of the input space. At the physical layer, the use of neural models operating directly on signal representations follows the research direction described by O’Shea and Hoydis [32].

These considerations motivate the use of the convolutional autoencoder and OC-SVM as reference detectors in the present study. Both operate under the same one-class assumption and are trained exclusively from jamming-free observations. Their different internal mechanisms—reconstruction error for the CAE and nominal-support estimation for the OC-SVM—provide complementary reference points for assessing whether a sensitivity loss is specific to one detection strategy.

A separate issue concerns how detector performance should be evaluated. Receiver operating characteristic curves and the corresponding area under the curve are standard tools for measuring class separability [33,34]. Statistical comparisons between ROC curves can be performed using methods such as that of DeLong et al. [35], while bootstrap procedures provide a general framework for estimating sampling variability [36]. These tools remain valuable and are retained in the present study.

However, AUC and operational sensitivity answer different questions. AUC integrates separability over the full threshold range and therefore includes false-alarm probabilities that may not correspond to the intended operating regime of a physical-layer monitor. Moreover, AUC is dimensionless and does not indicate how much jammer power is required before a detector becomes operationally reliable. A detector can consequently exhibit a high AUC and still perform poorly at a restrictive false-alarm operating point.

Classical detection theory instead characterizes sensitivity with respect to a prescribed detection and false-alarm requirement [37]. Following this principle, the present framework fixes $P_{\mathrm{FA}}=10^{-2}$ and determines the relative jammer level required to reach $P_{D}=0.9$, defining the detection sensitivity floor $L_{0.9}^{★}$. The communication side supplies a second reference, $L_{0.05}^{\mathrm{SER}}$, corresponding to the relative jammer level at which the preamble reaches the selected degradation criterion. The separation between these two quantities defines the early-warning margin. Accordingly, AUC measures overall statistical separability, $L_{0.9}^{★}$ measures operational detection sensitivity, and $M_{0.9}$ relates that sensitivity to the onset of measurable preamble degradation.

A complementary line of research addresses jamming recognition under limited labeled data or previously unseen attack classes. Few-shot approaches reduce the amount of labeled jamming data required during training; for example, Luo et al. [38] proposed a time–frequency self-attention and knowledge-distillation framework for few-shot radar jamming recognition, while Ding et al. [39] combined conditional generative data augmentation with a fusion CNN to improve jamming recognition when only a small number of labeled examples are available. Open-set and open-world approaches address a related but distinct problem by explicitly allowing unknown jammer classes at test time. Zhang et al. [40] used a residual convolutional autoencoder within a zero-shot/open-world recognition framework, and Zhou et al. [41] proposed a sustainable open-set learning strategy that combines unknown-class recognition with similarity learning and pseudo-sample generation. More recently, Li et al. [42] addressed open-set communication-jamming recognition directly from raw I/Q observations using domain adaptation and adaptive thresholding. These approaches complement the nominal-only one-class setting adopted in the present work: whereas few-shot and open-set methods exploit limited labeled attacks or previously learned jammer classes to identify known and unknown attack types, the CAE and OC-SVM considered here are trained exclusively from jamming-free observations. Nevertheless, any such recognition method that produces a packet-level attack score could, in principle, be evaluated using the same sensitivity-floor and pre-degradation-margin framework proposed here.

### 2.6. Positioning

The present work lies at the intersection of LoRa physical-layer security, preamble processing, impairment analysis, and anomaly detection, but it addresses a question that none of these lines resolves independently. Prior work has established that LoRa jamming is practical and disruptive [3,4,5,10], that jamming can be detected from both higher-level and physical-layer observations [15,16,17], and that noise, fading, interference, and synchronization impairments alter LoRa reception [6,7,22,23,25]. What remains unresolved is how these two sides of the problem relate quantitatively: how much jammer-power separation exists between reliable detection and the selected preamble-degradation onset.

The early-warning margin addresses this gap by placing a detector-side quantity, $L_{0.9}^{★}$, and a communication-side quantity, $L_{0.05}^{\mathrm{SER}}$, on the same relative jammer-power axis. This makes heterogeneous monitors directly comparable in a physical unit and distinguishes early-warning capability from conventional measures of eventual detection or threshold-independent separability. A positive margin identifies a region in which the selected detection requirement is satisfied before the degradation criterion is reached, whereas a negative margin identifies a detector that becomes reliable only after that onset.

The dechirp-domain PMR statistic provides a complementary diagnostic perspective. Because it is derived from spectral concentration after a transformation matched to the LoRa chirp structure, it provides a physically interpretable reference against which the learned detectors can be evaluated. Its purpose is therefore not only to introduce another detector, but also to determine whether attack-related evidence remains accessible in the received preamble when a learned detector exhibits a pronounced loss of sensitivity. The contrasting behavior of PMR and the learned detectors across interference and fading conditions further motivates the fusion strategy evaluated later in the paper.

Finally, the severity-graded composite-channel ladder examines whether sensitivity trends identified under isolated impairments persist when noise, multipath, co-channel interference, and synchronization impairments act jointly. This extension is important because isolated conditions are useful diagnostic tools but do not reproduce the simultaneous impairment structure of a deployment channel. The composite analysis therefore evaluates the transferability of isolated-condition conclusions rather than assuming that individual impairment effects combine independently. As a complementary robustness check, we additionally examine how fixed learned detectors and source-derived operating thresholds respond when a single physical-layer parameter is changed while the remaining nominal conditions are preserved, without target-domain recalibration.

To the best of our knowledge, previous LoRa jamming studies have not jointly formulated detector sensitivity and preamble degradation as a decibel-valued early-warning margin on a common relative jammer-power axis. The present framework complements prior early-detection work by quantifying not only whether jamming can be recognized before the selected preamble-degradation onset, but how much jammer-power headroom separates reliable detection from that onset.

## 3. Problem Formulation and Evaluation Framework

### 3.1. Signal and Threat Model

Let x[n] denote a discrete-time complex baseband LoRa packet with spreading factor SF, bandwidth *B*, and sampling rate $F_{s}$. Under a generic LoRa physical-layer configuration, one LoRa symbol spans

(1) $$N=2^{\mathrm{SF}}\frac{F_{s}}{B}.$$

More generally, a monitoring window comprising $M_{p}$ complete preamble upchirps contains $L_{w}=M_{p}N$ complex samples. The experimental instantiation considered in this study uses SF=7, B=125 kHz, $F_{s}=250$ kHz, and $M_{p}=8$, yielding N=256 samples per symbol and $L_{w}=2048$ samples. The monitored interval therefore corresponds to the first eight nominal preamble upchirps and excludes the start-frame delimiter, header, and payload.

The received observation is modeled as

(2) $$y\left[n\right]=e^{\;j2\pi \Delta fn/F_{s}}\left[\left(h_{s}*x\right)\left[n\right]+\alpha_{c}\left(h_{i}*d_{\Delta }\left\{x_{i}\right\}\right)\left[n\right]\right]+j_{L}\left[n\right]+w\left[n\right],$$

where $h_{s}$ and $h_{i}$ denote the channel impulse responses of the desired and interfering branches, respectively; $\alpha_{c}$ scales a co-channel LoRa interferer $x_{i}\left[n\right]$ delayed through $d_{\Delta }\left\{\cdot \right\}$; Δf is the effective carrier-frequency offset; w[n] is additive white Gaussian noise; and $j_{L}\left[n\right]$ denotes the injected jammer waveform. In the isolated co-channel conditions, the desired branch is taken as ideal and the interfering branch is represented by a single complex flat-fading coefficient, following the standard narrowband approximation for uncorrelated co-channel transmissions [43].

The model in Equation (2) provides a common abstraction for the impairment families considered in this study rather than implying that all terms are active simultaneously in every experiment. In the isolated-impairment conditions, only the term associated with the impairment under evaluation is enabled, while the remaining components are suppressed or fixed to their nominal values. In contrast, the composite-channel conditions activate several impairment mechanisms jointly. The signal $x_{i}\left[n\right]$ denotes an independently generated co-channel LoRa transmission, whereas $d_{\Delta }\left\{\cdot \right\}$ represents its relative temporal displacement with respect to the desired packet. The jammer waveform $j_{L}\left[n\right]$ is introduced independently of the co-channel interferer and is scaled after normalization of the desired waveform, allowing its relative level *L* to be swept in a controlled manner without changing the nominal amplitude reference of the victim signal. This formulation therefore separates propagation, carrier-frequency offset, co-channel interference, and intentional jamming within a common received-signal model while allowing each contribution to be examined either independently or in combination. The discrete sampling-time offset considered in the synchronization scenarios is specified separately in Section 5.

The formulation itself is not intrinsically restricted to the numerical LoRa PHY configuration adopted in the experiments. Alternative spreading factors, bandwidths, sampling rates, and monitored preamble lengths can be represented through the corresponding waveform and sampling parameters, without changing the definitions of the detection sensitivity floor or the pre-degradation margin introduced below. The quantitative detector sensitivities and margins reported in this work, however, remain specific to the evaluated SF=7, B=125 kHz configuration and should not be assumed to transfer unchanged to other LoRa PHY settings.

The adversary transmits a LoRa-like waveform concurrently with the victim packet and begins at sample $n_{0}=20$, inside the first observed preamble symbol. The attack therefore requires neither payload knowledge nor successful demodulation of the victim transmission. This fixed preamble-directed attack model is intentionally retained throughout the study so that the relative jammer level can be varied independently of the receiver-side impairment conditions. It therefore provides a controlled sensitivity benchmark for the considered LoRa-like jammer rather than an exhaustive representation of reactive, adaptive, frequency-selective, or protocol-aware jamming strategies.

Before jammer injection, the desired packet is power-normalized. The jammer amplitude is then selected so that its RMS level relative to the normalized desired signal satisfies

(3) $$L=20\log_{10}\left(\frac{\mathrm{RMS}\{j_{L}\}}{\mathrm{RMS}\{x\}}\right),$$

with *L* swept over

{−10,−9,…,+6}dB.

Equivalently, the RMS-amplitude definition above corresponds to a nominal digital jammer-to-signal power ratio expressed in decibels. Because this quantity is defined from the digitally generated and normalized waveforms, it must not be interpreted as a calibrated radio-frequency power level such as dBm or dBW.

### 3.2. Operating-Characteristic Figures of Merit

Let s(·) denote any packet-level anomaly score, oriented so that larger values correspond to stronger evidence of jamming, and let $D_{0}$ denote the independent nominal, jamming-free calibration partition associated with the channel condition under evaluation. This calibration partition is mutually disjoint from the training, validation, and held-out clean test partitions described in Section 5. For every detector, the operating threshold is obtained from the empirical 99th percentile of its calibration scores,

(4) $$\tau ={\hat{Q}}_{0.99}\left(\{s\left(y\right):y\in D_{0}\}\right)$$

This calibration targets a nominal false-alarm probability of

$$P_{\mathrm{FA}}=10^{-2},$$

thereby placing all detectors at the same prescribed operating point. The realized false-alarm probability is subsequently estimated on the mutually independent held-out clean test partition; test observations do not contribute to preprocessing, model fitting, validation, or threshold selection. The detection probability at relative jammer level *L* is then

(5) $$P_{D}\left(L\right)=\mathrm{Pr}\left\{s\left(y\right)>\tau |L\right\},$$

estimated empirically from jammer-injected observations generated only from the frozen held-out test realizations at that level. The pair $\left(P_{D},P_{\mathrm{FA}}\right)=\left(0.9,10^{-2}\right)$ is used as a common operating convention rather than as a protocol-specific requirement. It imposes a high detection-probability target while restricting nominal false alarms to one percent, and, most importantly for the present framework, provides a fixed reference at which heterogeneous detectors can be compared. Alternative operating points would shift the absolute sensitivity floors but would not alter the definition of the framework.

#### 3.2.1. Detection Sensitivity Floor

For a target detection probability δ, we define the detection sensitivity floor as

(6) $$L_{\delta }^{★}=\min\left\{L\in \left[L_{\min},L_{\max}\right]:P_{D}\left(L\right)\geq \delta \right\}.$$

The crossing is estimated by linear interpolation between the two adjacent jammer levels that bracket the target probability. If multiple crossings occur as *L* increases, the first crossing is retained, consistent with the definition of the sensitivity floor as the earliest relative jammer level at which the prescribed detection criterion is reached. Throughout this work,

δ=0.9,

so that $L_{0.9}^{★}$ denotes the smallest relative jammer level within the evaluated sweep at which the interpolated detection characteristic reaches $P_{D}=0.9$ while operating at the prescribed $P_{\mathrm{FA}}=10^{-2}$.

If the target probability is not reached within the evaluated sweep, the sensitivity floor is right-censored and reported as not attained. Conversely, when the target criterion is already satisfied at the lowest evaluated jammer level, the floor is left-censored. Thus, when the target criterion is already satisfied at the lower sweep boundary, the sensitivity floor is reported as

$$L_{0.9}^{★}\leq -10.00\;\mathrm{dB},$$

indicating that the actual crossing may lie below the evaluated jammer range.

#### 3.2.2. Link-Degradation Onset

A communication-side reference is obtained directly from the monitored preamble. Each of the eight observed upchirps is dechirped using the conjugate of an ideal reference chirp and transformed to the frequency domain. The dominant spectral bin is taken as the detected preamble symbol and compared with the corresponding bin obtained from the matched jamming-free realization.

Let SER(L) denote the resulting mean preamble symbol error rate at jammer level *L*. The preamble-based link-degradation onset is defined as

(7) $$L_{0.05}^{\mathrm{SER}}=\min\left\{L:\mathrm{SER}\left(L\right)-{\mathrm{SER}}_{0}\geq 0.05\right\},$$

where ${\mathrm{SER}}_{0}$ is the jamming-free preamble SER under the same channel condition. Thus, $L_{0.05}^{\mathrm{SER}}$ identifies the first relative jammer level at which the preamble SER increases by five percentage points with respect to its own nominal baseline. The five-percentage-point increase is adopted as a practical onset criterion rather than as a universal LoRa link-failure threshold. Its purpose is to identify a reproducible departure from the jamming-free preamble baseline while preserving sensitivity to the initial degradation induced by the jammer. A substantially stricter criterion could become dominated by the nominal variability of challenging propagation conditions, whereas a much larger increment would shift the reference toward a more advanced stage of preamble deterioration. Accordingly, $L_{0.05}^{\mathrm{SER}}$ should be interpreted as the onset of measurable jammer-induced preamble degradation under the selected criterion, rather than as the point of complete packet-acquisition failure. The sensitivity of the resulting onset to the selected SER increment is examined in Appendix B.

The onset is estimated using the same first-crossing linear interpolation applied to the detection sensitivity floor, replacing $P_{D}\left(L\right)$ with $\mathrm{SER}\left(L\right)-{\mathrm{SER}}_{0}$ and the target probability with the 0.05 degradation criterion. Using the SER increment rather than an absolute error-rate threshold is important because some propagation conditions already introduce nonzero preamble errors before jamming is applied. Under the severe fading condition, for example,

$${\mathrm{SER}}_{0}=0.124.$$

An absolute SER criterion would therefore confound degradation produced by the propagation channel with degradation caused by the jammer. Referencing each condition to its own jamming-free baseline isolates the additional degradation associated with the attack.

#### 3.2.3. Early-Warning Margin

The early-warning margin is defined as

(8) $$M_{0.9}=L_{0.05}^{\mathrm{SER}}-L_{0.9}^{★}.$$

A positive margin indicates a jammer-power interval over which the detector has already reached the prescribed $\left(P_{D},P_{\mathrm{FA}}\right)$ operating point while the preamble has not yet crossed the selected degradation criterion. A negative margin indicates that the detector reaches the required detection probability only after that criterion has been exceeded. Accordingly, the margin quantifies early warning in the jammer-power domain rather than as elapsed time.

Censoring of the sensitivity floor propagates directly to the margin. In particular, when

$$L_{0.9}^{★}\leq -10\;\mathrm{dB},$$

the corresponding early-warning margin is lower-bounded rather than known exactly. For example, a reported boundary value obtained from such a floor must be interpreted as

$$M_{0.9}\geq L_{0.05}^{\mathrm{SER}}-\left(-10\;\mathrm{dB}\right).$$

If the sensitivity floor is not attained within the upper limit of the sweep, a finite early-warning margin cannot be assigned within the evaluated jammer range.

Figure 1 places the three quantities on the same relative jammer-level axis.

### 3.3. Why a Decibel Margin

Three properties motivate the use of a decibel-valued margin.

First, it is commensurable. Detectors with fundamentally different architectures and anomaly scores are mapped onto the same physical axis through the relative jammer level required to satisfy a common $\left(P_{D},P_{\mathrm{FA}}\right)$ criterion. The comparison therefore does not depend on the numerical scale of reconstruction errors, kernel decision functions, or spectral-concentration scores.

Second, it is operationally interpretable. The margin directly represents jammer-power headroom between reliable detection and the selected preamble-degradation onset. Because a difference of $M_{0.9}$ decibels corresponds to a jammer-power ratio of

(9) $$10^{M_{0.9}/10},$$

an 11dB margin corresponds to a jammer-power ratio of approximately 12.6 between the two operating points, whereas a 1dB margin corresponds to a ratio of approximately 1.26. The metric therefore distinguishes a broad pre-degradation detection region from one in which detection and degradation occur at nearly the same jammer level. This interpretation concerns jammer-power headroom and does not, by itself, imply a particular elapsed warning time.

Third, it is diagnostic. For a fixed channel and attack condition, $L_{0.05}^{\mathrm{SER}}$ is independent of the monitoring algorithm. Differences in $M_{0.9}$ across detectors therefore arise from differences in their sensitivity floors. This permits detector-dependent sensitivity losses to be distinguished from conditions in which discriminative attack evidence becomes weak for multiple monitoring strategies, a distinction examined in Section 6.

### 3.4. Interpretation and Scope of the Framework

The proposed framework deliberately separates three questions that are often conflated in detector evaluation. The area under the ROC curve characterizes the overall statistical separability between nominal and jammed observations across the complete threshold range; the detection sensitivity floor $L_{0.9}^{★}$ identifies the relative jammer level at which a detector satisfies the prescribed $\left(P_{D},P_{\mathrm{FA}}\right)$ operating requirement; and the early-warning margin $M_{0.9}$ determines whether that level is reached before or after the selected preamble-degradation onset. These quantities are therefore complementary rather than interchangeable. In particular, two detectors with similar AUC may exhibit substantially different sensitivity floors, and a detector with strong overall separability may still provide a small or even negative early-warning margin at the selected operating point.

The early-warning margin should be interpreted as a power-domain measure of pre-degradation detectability. It does not represent elapsed warning time, nor does it assume a particular temporal evolution of jammer power. Rather, it quantifies the relative jammer-power interval separating reliable detection from the selected preamble-degradation criterion. A larger positive value therefore indicates greater sensitivity headroom before measurable degradation is observed, whereas a negative value indicates that the detector becomes reliable only after the selected degradation onset has been crossed.

Likewise, $L_{0.05}^{\mathrm{SER}}$ is intentionally defined from the monitored preamble rather than from payload decoding, packet error rate, or end-to-end service failure. The framework therefore does not claim that $L_{0.05}^{\mathrm{SER}}$ represents complete loss of communication. Instead, it provides a reproducible communication-side reference tied to the same signal region used for physical-layer monitoring. This choice keeps the detector-side and communication-side quantities directly comparable and allows both to be estimated from the same relative jammer-level sweep.

Finally, the framework is detector-agnostic. Any monitoring method that produces a packet-level score can be mapped to a sensitivity floor under a prescribed $\left(P_{D},P_{\mathrm{FA}}\right)$ operating point and, when a corresponding preamble-degradation reference is available, to a pre-degradation margin. Here, detector-agnostic refers to the evaluation layer rather than to a particular detector architecture or training paradigm. The framework can accommodate fixed, adaptive, or online monitoring methods, provided that they produce a packet-level score and a corresponding detection characteristic from which the prescribed operating point can be determined. The detector implementations evaluated in the present study, however, remain fixed after model development and operating-threshold calibration. The proposed formulation is therefore intended as an evaluation framework rather than as a metric tied specifically to the CAE, OC-SVM, or PMR detectors considered in this study.

## 4. A Dechirp-Domain Sensitivity Statistic

### 4.1. Spectral Concentration After Dechirping

Standard LoRa reception exploits the deterministic chirp structure by multiplying the received signal by the conjugate of a reference upchirp and transforming the resulting sequence to the frequency domain [18,19,20]. Under ideal alignment, dechirping converts a nominal LoRa upchirp into an approximately single-frequency complex exponential, so that its discrete Fourier transform concentrates most of the spectral magnitude around one dominant bin.

Let $r_{m}\left[n\right]$ denote the *m*-th preamble symbol within the observation window, m=1,…,8. For each symbol, we compute

(10) $$R_{m}\left[k\right]=\sum_{n=0}^{N-1}r_{m}\left[n\right]\;c^{*}\left[n\right]\;e^{-j2\pi kn/N},\;k=0,\ldots ,N-1,$$

where c[n] is the ideal reference upchirp and N=256. Deviations from the nominal chirp structure modify the resulting spectral concentration. Additive noise and multipath can spread energy across multiple bins, while an additional LoRa-like signal with a different relative shift can introduce competing spectral components. These effects generally alter the concentration of spectral magnitude relative to the corresponding nominal condition. Across most of the operating regimes considered here, the jammer reduces dominance of the nominal spectral peak, although the direction of this change can reverse under very low-SNR conditions, as discussed in the following subsection.

Building on the standard LoRa dechirp-and-frequency-domain processing described in [18,19,20], we define the following packet-level dechirp-domain peak-to-mean ratio:

(11) $$\Pi =\frac{1}{8}\sum_{m=1}^{8}\frac{\max_{k}\left|R_{m}\left[k\right]\right|}{\frac{1}{N}\sum_{k=0}^{N-1}\left|R_{m}\left[k\right]\right|}$$

Averaging the per-symbol ratios over the eight preamble upchirps produces a single packet-level statistic aligned with the observation window used by the reference detectors. It also prevents the decision from depending on the concentration behavior of a single preamble symbol, while preserving the deterministic acquisition structure targeted by the jammer. For each symbol, the ratio compares the largest spectral magnitude with the mean magnitude across all *N* bins. Because

$$\max_{k}|R_{m}\left[k\right]|\leq \sum_{k=0}^{N-1}\left|R_{m}\left[k\right]\right|,$$

the per-symbol ratio, and consequently Π, is bounded above by *N*. For the present configuration,

0<Π≤256.

The upper bound is approached when essentially all spectral magnitude is concentrated in a single bin. As a numerical consistency check, the quasi-ideal reference condition considered in this study yields Π=255.99, confirming both the expected limiting behavior and the implementation of the statistic.

Two properties make Π particularly suitable as a monitoring statistic. First, it is invariant to common amplitude scaling: multiplication of the received symbol by a constant magnitude scales both the numerator and denominator equally and therefore leaves the ratio unchanged. A common phase rotation likewise does not affect the statistic because it is computed from spectral magnitudes. Consequently, PMR does not require an absolute received-power calibration.

Second, PMR is directly linked to operations already associated with LoRa acquisition. When the dechirped frequency-domain representation is available, the additional operations required by the statistic reduce to identifying a maximum, computing a mean, and forming one ratio per observed preamble symbol, followed by averaging across the eight upchirps. PMR therefore provides a low-dimensional and physically interpretable alternative to detectors operating directly on the full three-channel preamble representation.

### 4.2. One-Class Test

The PMR detector follows the same nominal-only calibration principle used throughout the evaluation framework. Across most of the operating regimes considered here, the addition of the jammer reduces the spectral concentration relative to the corresponding jamming-free distribution. The resulting test is therefore defined on the lower tail of Π.

To preserve the common convention that larger anomaly scores indicate stronger evidence of jamming, we define

(12) $$s_{\mathrm{PMR}}=-\Pi .$$

Applying Equation (4) to this score is equivalent to declaring a packet anomalous when

(13) $$\Pi <{\hat{Q}}_{0.01}\left(\{\Pi \left(y\right):y\in D_{0}\}\right).$$

The threshold is therefore estimated independently from the jamming-free PMR distribution associated with each channel condition. No jammed observations are required for its calibration, and no universal PMR threshold is assumed across different impairment regimes.

The lower-tail formulation intentionally encodes a directional hypothesis: jamming is expected to reduce concentration. This hypothesis is physically appropriate across most evaluated conditions but is not universal. At very low signal-to-noise ratio, the nominal dechirped spectrum can already be highly diffuse, while the addition of a LoRa-like jammer may introduce a sufficiently structured spectral component to increase, rather than decrease, the dominant-bin concentration. In this regime the orientation of the statistic can invert and the one-sided detector loses power.

A two-sided PMR test could in principle respond to deviations in either direction. Such a test would, however, require a different calibration rule that allocates the prescribed false-alarm probability across the two tails. We retain the lower-tail formulation because it preserves a single physically interpretable hypothesis and a direct correspondence with the nominal 1st-percentile threshold. The low-SNR inversion is therefore treated explicitly as a limitation of the proposed statistic rather than hidden through condition-dependent changes in the decision rule.

### 4.3. Reference Detectors

Two learned one-class detectors are retained as reference comparators: a CAE and a OC-SVM. Their role in the present study is not to introduce new detector architectures, but to provide learned baselines with fundamentally different anomaly-scoring mechanisms against which the dechirp-domain statistic and the early-warning framework can be evaluated.

Both detectors operate on the same packet representation,

(14) $$X\in R^{3\times 2048},$$

formed by stacking the in-phase component *I*, the quadrature component *Q*, and the magnitude

|I+jQ|

over the complete eight-upchirp observation window. Both models are trained exclusively from jamming-free packets, preserving the one-class assumption used throughout the study. Model fitting and preprocessing use only the clean training partition, which is mutually disjoint from the validation, calibration, and held-out test partitions described in Section 5.

The convolutional autoencoder applies a depthwise one-dimensional convolution with kernel size 5 independently to the three input channels, followed by a conventional convolution that combines information across channels. Fully connected layers project the resulting representation to a 16-dimensional latent code, after which the decoder mirrors the encoding operation to reconstruct the original input. Intermediate layers use LeakyReLU activations with negative slope 0.01, while the output layer is linear.

Training minimizes the mean squared reconstruction error with Adam at learning rate $10^{-3}$ and batch size 32. A mutually disjoint clean validation partition is used exclusively for autoencoder training monitoring and does not contribute to model fitting or operating-threshold calibration. Training is performed for 30 epochs in the general scenarios and for 10 epochs in the synchronization-impairment scenarios. The anomaly score is the reconstruction error.

(15) $$s_{\mathrm{CAE}}=\frac{1}{\left|X\right|}{∥X-\hat{X}∥}_{2}^{2},$$

where |X|=3×2048=6144 denotes the number of scalar elements in the input representation, so that larger values of $s_{\mathrm{CAE}}$ indicate increasing deviation from the nominal signal structure learned during training.

The OC-SVM uses the same three-channel input but flattens it into a vector in

$$R^{6144}.$$

The features are standardized using statistics fitted exclusively on the nominal training partition. Neither the clean calibration partition nor the held-out clean test partition contributes to feature standardization or OC-SVM fitting. The resulting vectors are processed by a radial basis function kernel with γ determined by the scale heuristic and ν=0.01 [30]. Since the conventional OC-SVM decision function assigns larger values to observations lying within the estimated nominal support, its sign is reversed to maintain the common anomaly-score orientation,

(16) $$s_{\mathrm{OCSVM}}=-f_{\mathrm{OCSVM}}\left(X\right),$$

where larger values again correspond to stronger evidence of deviation from the jamming-free distribution.

Using a common input observation and nominal-only training assumption allows the reference detectors to be compared under a consistent experimental basis, while their different scoring mechanisms provide complementary responses to the physical-layer impairments considered later in the study. For both learned detectors, the operating threshold is subsequently estimated exclusively from the independent clean calibration partition according to the procedure defined in Section 3.2 and detailed in Section 5.

### 4.4. Fusion Rule

The CAE reconstruction error and the PMR statistic respond to different properties of the received preamble. The former measures consistency with learned nominal reconstruction behavior, whereas the latter measures spectral concentration after a transformation explicitly matched to the LoRa chirp structure. Their different sensitivities across impairment regimes motivate a deliberately minimal score-level fusion rule.

The fusion analysis is intentionally restricted to CAE and PMR because its purpose is to test the complementarity between reconstruction-based evidence and a physically matched dechirp-domain statistic, rather than to optimize a multi-detector ensemble. The OC-SVM is retained as an independent learned reference throughout the evaluation.

Because the two scores have unrelated numerical scales, each is first standardized using robust statistics estimated exclusively from the corresponding clean training partition,

(17) $$\tilde{s}=\frac{s-\mathrm{med}(s)}{\mathrm{IQR}(s)/1.349},$$

where med(·) denotes the median and IQR(·) the interquartile range. The factor 1.349 maps the interquartile range to the standard-deviation scale for a Gaussian reference distribution, while retaining the robustness of quantile-based normalization.

The fused score is then defined as the pointwise maximum,

(18) $$s_{\mathrm{fus}}=\max\left({\tilde{s}}_{\mathrm{CAE}},{\tilde{s}}_{\mathrm{PMR}}\right).$$

This rule can be interpreted as retaining, for each packet, the stronger standardized anomaly evidence supplied by either detector. It introduces no learned fusion weights or tunable combination parameters and does not require impairment-specific switching between the CAE and PMR scores at decision time, while preserving a single scalar output compatible with the framework of Section 3. This property concerns the fusion decision itself and should not be interpreted as online adaptation of the constituent detectors or their operating-point calibration.

Importantly, the fused detector does not inherit the individual thresholds of its constituent scores. Instead, Equation (4) is applied again to the nominal fused-score distribution itself. The resulting threshold is therefore calibrated directly at the prescribed nominal false-alarm probability,

$$P_{\mathrm{FA}}=10^{-2},$$

rather than obtained by combining two independently thresholded decisions. This preserves a common operating-point definition for the CAE, OC-SVM, PMR, and fused detector.

## 5. Experimental Design

### 5.1. Waveform Generation

Reference packets were generated using the GNU Radio LoRa physical-layer implementation of Tapparel and Burg [21], configured with spreading factor SF=7, bandwidth B=125 kHz, coding rate 4/5, sampling rate $F_{s}=250$ kHz, cyclic redundancy check enabled, and implicit header mode. The selected SF=7, B=125 kHz configuration is consistent with standardized LoRaWAN physical-layer operating configurations defined in the LoRa Alliance Regional Parameters specification [44]. It was retained as a fixed reference configuration throughout the study to preserve a common waveform, sampling structure, and observation length across all impairment campaigns, thereby allowing detector sensitivity differences to be attributed to the evaluated channel and interference conditions rather than to changes in the underlying LoRa PHY configuration.

Independent jamming-free populations were generated for model training, validation, operating-threshold calibration, and held-out testing, as detailed in Section 5.4. These partitions are defined at the clean-realization level before any jammer injection and are mutually disjoint. For the isolated-condition campaigns, the held-out evaluation population consists of 2000 clean test realizations with traceable payloads.

All jammer-injected test observations are subsequently derived from deep copies of this frozen held-out test population. The same underlying clean test realization is intentionally reused across relative jammer levels, so that comparisons along the jammer-power sweep are paired at the clean-realization level while remaining completely isolated from model development and operating-threshold calibration.

For every condition, the first 2048 complex samples corresponding to the eight nominal preamble upchirps were retained as the monitoring window described in Section 3. The jammer was injected according to the threat model of Equation (2), with its relative level swept over the common 17-point grid

L∈{−10,−9,…,+6}dB.

Using the same jammer-level grid across conditions allows detection sensitivity floors and, where the corresponding preamble-degradation reference is available, early-warning margins to be evaluated on a common relative-power axis.

### 5.2. Isolated Impairment Conditions

The isolated experiments activate one impairment family at a time in order to identify how each physical mechanism modifies detector sensitivity independently of the others. Four families are considered: additive white Gaussian noise (AWGN), severe multipath fading, co-channel LoRa interference, and synchronization impairments combining carrier-frequency and sampling-time offsets. Their configurations are summarized in Table 1.

The AWGN family comprises four signal-to-noise ratios, while the co-channel and CFO/STO families each comprise four operating configurations. Together with the single severe-fading profile, these yield 13 isolated channel configurations. Each configuration is evaluated over the same 17 jammer levels, producing 221 isolated channel–jammer operating points.

The co-channel level is defined relative to the desired LoRa signal, while the CFO entries in Table 1 denote the nominal offset $\Delta f_{0}$ around which packet-to-packet Gaussian frequency variability is introduced. Sampling-time offset is independently drawn over the stated discrete range. These variations are represented within the nominal distribution associated with each synchronization condition and are therefore also present during one-class detector training and threshold calibration.

A quasi-ideal line-of-sight profile with negligible dispersion was additionally retained as a numerical reference condition. It is used only to verify the limiting behavior of the dechirp-domain statistic, including the upper bound of Π, and is excluded from detector comparisons because a corresponding OC-SVM campaign was not available.

### 5.3. Severity-Graded Composite Conditions

The isolated conditions are diagnostic by construction: they reveal the effect of individual impairment mechanisms but do not reproduce the simultaneous distortions expected in a deployed link. We therefore define a three-step severity-graded composite ladder in which AWGN, multipath propagation, co-channel interference, carrier-frequency offset, packet-to-packet frequency variability, and sampling-time offset act jointly.

The severity-graded composite conditions are summarized in Table 2. The ladder is a controlled progression rather than a random draw from a channel ensemble. The ladder is a controlled progression rather than a random draw from a channel ensemble. Impairment severity is non-decreasing across the listed dimensions from C1 to C3, with at least one relevant impairment becoming strictly more adverse at each step. C1 represents the mildest jointly impaired condition; C2 increases co-channel interference while retaining the remaining nominal parameters of C1; and C3 further reduces SNR, strengthens co-channel interference, increases the nominal CFO, and replaces the mild Rician propagation profile with a severe Rayleigh multipath channel. This construction therefore provides a monotonic severity ladder while avoiding the artificial requirement that every individual parameter change at every step.

The ladder is intentionally severity-graded rather than statistically representative of a particular deployment environment. Its purpose is to determine whether detector sensitivity degrades consistently as jointly applied impairments become progressively more adverse and whether detector rankings established under isolated conditions remain valid when the same mechanisms interact. The controlled monotonic progression therefore provides a direct stress test of the transferability of isolated-impairment conclusions, while no extrapolation beyond the three evaluated composite levels is implied.

### 5.4. Protocol, Estimation and Statistical Procedures

For each impairment condition, mutually independent jamming-free training, validation, calibration, and test partitions were generated before model fitting or jammer injection. In the AWGN, severe-fading, CCI, and CFO/STO experiments, these partitions contained 1600, 400, 500, and 2000 clean realizations, respectively. The composite experiment followed the same separation principle and was conducted over four independent runs; each run contained 800 training, 200 validation, 500 calibration, and 500 held-out test realizations. The complete dataset architecture is summarized in Table 3.

Partition independence was enforced at the underlying clean-realization level using disjoint packet identities and independent random-seed namespaces. Clean-side realization identifiers were assigned before jammer injection, and the training, validation, calibration, and test partitions were mutually disjoint. Preprocessing parameters and detector models were fitted using training data only; validation data were used only for autoencoder training monitoring, and the 99th-percentile operating thresholds were computed exclusively from the corresponding clean calibration partition.

After the clean test partition had been completely isolated and frozen, jammer-injected observations were generated only from deep copies of those test realizations. The same clean test realization was intentionally reused across relative jammer levels to enable paired evaluation of jammer strength, but no test realization was used for preprocessing, model fitting, validation, or threshold calibration. Both learned detectors were evaluated using the same held-out test realizations.

The frozen clean test partition was used to estimate the realized held-out false-alarm probability and provided the nominal class for AUC estimation, whereas the corresponding jammer-injected test observations were used to estimate detection probability and the jammer-power sensitivity floor and provided the attack class for AUC estimation. Thus, neither nominal test observations nor their jammed counterparts contributed to preprocessing, model fitting, validation, or operating-threshold selection. For the PMR statistic, which requires no model fitting, the operating threshold was likewise obtained exclusively from the clean calibration partition. For the score-level fusion, nominal standardization parameters were estimated from training data only, and the fused operating threshold was subsequently obtained from the independent clean calibration partition.

For each composite condition, the four independent runs yielded an aggregate held-out evaluation population of 2000 clean test realizations. Within each run, 500 clean held-out test realizations and their corresponding jammer-injected copies were evaluated at every relative jammer level using the operating threshold derived from that run’s independent clean calibration partition. Detector probabilities and score-based metrics were first computed from the packet-level observations within each run and were subsequently aggregated across the four independent runs. Accordingly, mean curves and their associated 95% confidence intervals were obtained from the run-level results rather than by treating the pooled packet-level observations as independent experimental replicates. This preserves independent model-development, calibration, and test populations within each run while accounting for between-run variability in the composite analysis.

Partition independence was verified using clean-side realization identifiers and pre-jammer I/Q information. All cross-partition intersections among training, validation, calibration, and test realizations were zero in the evaluated campaigns. Reusing the same frozen test realization across jammer levels was intentional and provided a paired evaluation of jammer strength without introducing information from the test partition into model development.

The complete campaign was additionally repeated over five physically independent realizations of every isolated condition, each with its own clean packets, channel and jammer draws, training, validation, calibration, and test partitions, and model fitting.

The isolated-condition tables and figures in Section 6.1, Section 6.2, Section 6.3, Section 6.4 and Section 6.5 report the first realization; the range of the sensitivity floor over the five realizations is reported next to the bootstrap intervals in Table 5. Across the thirteen isolated conditions of the first realization, the realized false-alarm probability on the held-out clean test partition ranged from 0.002 to 0.018 for the CAE and from 0.004 to 0.030 for the OC-SVM, against the nominal value of 0.01. For PMR, whose per-packet scores were re-exported for the fading, co-channel, and synchronization conditions of the same realizations, the realized false-alarm probability ranged from 0.004 to 0.015.

The 13 isolated channel configurations evaluated over 17 jammer levels produce

13×17=221

isolated channel–jammer operating points. The three composite configurations contribute an additional

3×17=51

operating points. For the primary point-estimate evaluation, each isolated operating point was evaluated using 2000 held-out jammer-injected test observations from the first physical realization. For the composite ladder, the four independent runs provided an aggregate of 2000 held-out jammer-injected test observations at each relative jammer level for each composite condition. These primary jammer sweeps therefore comprised

(221+51)×2000=544,000

jammed packet-level evaluations, in addition to the independent jamming-free populations used for training, validation, operating-threshold calibration, and held-out false-alarm evaluation. The additional physically independent repetitions of the isolated-condition campaigns used to characterize between-realization variability, as well as the controlled cross-condition calibration-transfer campaign of Section 5.5, are not included in this count. Accordingly, the value of 544,000 characterizes the primary evaluation population supporting the reported point estimates and should not be interpreted as the total number of packet-level evaluations performed across all supplementary repetition and robustness campaigns. For the composite campaign, statistical aggregation across repeated experiments is performed at the run level as described above.

Operating thresholds were obtained exclusively from the independent jamming-free calibration partition associated with the corresponding channel configuration and never from training, validation, held-out test, or jammed observations. The realized false-alarm probability was evaluated independently on the clean held-out test partition. For each detector and jammer level, the detection probability was estimated as the empirical fraction of jammer-injected held-out test observations exceeding the corresponding calibration-derived threshold.

Sensitivity floors were obtained from the first crossing of the prescribed $P_{D}=0.9$ criterion using the linear-interpolation procedure defined in Section 3. The same first-crossing interpolation principle was applied to the preamble-degradation onset whenever the corresponding SER reference was available.

Threshold-independent separability was additionally characterized through the area under the receiver operating characteristic curve. AUC was computed from the Mann–Whitney interpretation of the ROC area [34]. The nominal class used for AUC estimation was drawn exclusively from the held-out clean test partition, while the attack class was formed by the corresponding jammer-injected held-out test observations. Percentile bootstrap confidence intervals were obtained from 400 resamples of the held-out clean and jammer-injected test populations [36]. Across the isolated conditions, the resulting confidence intervals had a mean width of 0.010. These AUC estimates complement, rather than replace, the fixed-operating-point sensitivity measures introduced in Section 3.

Statistical uncertainty was additionally quantified for the three primary power-domain figures of merit: the detection sensitivity floor $L_{0.9}^{★}$, the preamble-degradation onset $L_{0.05}^{\mathrm{SER}}$, and the resulting power-domain early-warning margin $M_{0.9}$. For each detector and isolated channel condition, 2000 packet-level bootstrap replicates were generated. Within each replicate, the independent clean calibration partition was resampled with replacement and the corresponding 99th-percentile operating threshold was recomputed. The held-out test realization identifiers were then resampled with replacement using the same packet indices across the clean observation and all jammer levels, thereby preserving the paired structure of the jammer-power sweep.

For every bootstrap replicate, the complete detection characteristic $P_{D}\left(L\right)$ was recomputed over the 17 jammer levels, and the sensitivity floor was re-estimated using the same first-crossing and linear-interpolation procedure applied to the original point estimate. The jamming-free preamble-SER baseline and the jammer-induced SER increment were likewise recomputed from the resampled held-out test population, and the degradation onset was obtained from the first crossing of the 0.05 degradation criterion. The margin was then calculated within each bootstrap replicate as

(19) $$M_{0.9}^{\left(b\right)}=L_{0.05}^{\mathrm{SER},(b)}-L_{0.9}^{★,(b)}$$

For uncensored quantities, two-sided 95% confidence intervals were obtained from the 2.5th and 97.5th percentiles of the resulting bootstrap distributions. This procedure propagates finite-sample uncertainty from operating-threshold calibration and held-out packet evaluation through the first-crossing interpolation and margin calculation. Because the sensitivity floor and degradation onset are recomputed jointly within each replicate, the uncertainty of the resulting margin preserves the statistical dependence between its two constituent quantities.

When a crossing lay outside the evaluated jammer-power range, the corresponding quantity retained its one-sided censoring bound rather than being extrapolated beyond the tested sweep. Bootstrap replicates for which the crossing remained outside the sweep were treated as censored accordingly, and the proportion of censored replicates was retained as an additional indication of boundary uncertainty. The resulting intervals therefore quantify finite calibration- and held-out-test population uncertainty conditional on the fitted detector; they do not include detector-refitting variability or uncertainty associated with departures from the linear interpolation assumption between adjacent 1 dB jammer levels. The numerical sources and scripts used to regenerate the manuscript figures and tables, together with the packet-level detector scores and preamble-SER records used in the statistical analyses, are provided in Supplementary File S1 and Supplementary Data S1 and S2.

### 5.5. Controlled Cross-Condition Calibration Stress Test

To complement the condition-matched impairment-isolation and composite-channel experiments, an additional controlled cross-condition stress test was conducted to examine whether a detector and operating threshold calibrated under one jointly impaired nominal condition preserve their operating characteristics after a controlled change in the physical environment. The purpose of this auxiliary experiment is not to implement domain adaptation or online detector updating, but to quantify the sensitivity of fixed source-calibrated operating points to changes in nominal channel statistics. This auxiliary campaign is intended only to examine how the two learned reference detectors respond under such controlled calibration transfer. PMR and the score-level fusion are not included in this experiment; therefore, the results are interpreted as a diagnostic assessment of calibration transfer for the learned detectors rather than as an extension of the four-detector comparison presented in the main condition-matched analysis.

Unlike the isolated-impairment experiments, every clean realization in this campaign contains all four impairment mechanisms simultaneously: desired-link multipath fading, an independent delayed co-channel LoRa interferer, sampling- and carrier-frequency offsets, and additive white Gaussian noise. Each realization is generated by first applying multipath fading to the desired signal, then adding the independently faded and delayed co-channel interferer, applying STO and CFO to the resulting mixture, and finally adding AWGN. The monitoring window and LoRa PHY configuration follow Section 5.1, while both learned detectors retain the same IQMag representation used in the main evaluation. After extraction of the monitoring window, each packet is normalized independently by its maximum absolute value.

A single jointly impaired source condition is shared by all four transfer scenarios. It uses SNR=0 dB, the severe five-path Rayleigh desired-link profile defined in Section 5.2, CCI =−3 dB, nominal CFO =+1000 Hz, packet-wise Gaussian CFO jitter $\epsilon ∼N(0,50^{2})$ Hz clipped to ±150 Hz, and an independently generated integer STO uniformly distributed over {0,…,64} samples. The co-channel interferer is independently generated for each realization and is affected by an independent flat-Rayleigh coefficient and relative delay according to the established CCI model.

For each detector, the source-domain data are partitioned into 1600 clean training realizations, 400 clean validation realizations, and 500 clean calibration realizations. A separate set of 2000 independently generated clean realizations is reserved exclusively for evaluation under each shifted target condition. Source training, validation, calibration, and target-test populations are mutually disjoint. Independence was verified using realization identifiers and independent generation-seed namespaces, with zero cross-partition and source–target intersections. The CAE and OC-SVM retain the same architectures, preprocessing procedures, and training configurations defined in Section 4.3. No target-domain observation contributes to preprocessing, detector fitting, validation, or operating-threshold calibration.

For each detector, the source operating threshold is obtained from the independent clean calibration partition following the procedure defined in Section 3.2 and is then transferred unchanged to all shifted target conditions. No target-domain recalibration or detector adaptation is performed. The resulting target-domain false-alarm probability is estimated as

(20) $${\hat{P}}_{\mathrm{FA},\mathrm{target}}=\frac{1}{N_{t}}\sum_{i=1}^{N_{t}}I\left[s\;\left(y_{i}^{\mathrm{target}}\right)>\tau_{\mathrm{source}}\right],\;N_{t}=2000,$$

where $\tau_{\mathrm{source}}$ is the source-calibrated threshold. The resulting ${\hat{P}}_{\mathrm{FA},\mathrm{target}}$ is retained as the target-domain operating-point quantity reported in Section 6.7.

Four target scenarios are constructed by changing exactly one physical factor relative to the common source condition while retaining the remaining source parameters unchanged. In S1, only SNR changes from 0 to +8 dB. In S2, only the co-channel interference level changes from −3 to −9 dB. In S3, only the nominal CFO changes from +1000 to +500 Hz, while the CFO-jitter and STO distributions remain unchanged. In S4, only the desired-link fading law changes from the severe five-path Rayleigh profile to a five-path Rician channel with K=6 dB. This single-factor construction isolates the effect of each controlled physical-layer change while avoiding the simultaneous parameter variations considered in the composite-channel ladder.

For S4, the Rician target preserves the same physical-delay references {0,1.5,3.5,7,12} μs, discrete delays {0,0,1,2,3} samples, and relative path powers {0,−2.5,−5.5,−9,−13} dB as the severe-fading source profile. The path powers are normalized in linear power, the specular component is placed on the first tap, and its LOS phase is independently drawn from U[0,2π) for each packet. Both the source Rayleigh channel and the target Rician channel are implemented as independent packet-wise block fading, with a new set of path coefficients generated for each packet and held constant over the complete packet duration. Thus, no intra-packet temporal fading evolution or sample-wise Doppler process is applied.

After each clean target population was generated and frozen within each independent run, jammer-injected observations were produced exclusively from copies of those same realizations, following the same jammer model and relative-power sweep used in the main experiments. The complete set

L∈{−10,−9,…,+6}dB

was evaluated for every target condition. The same underlying clean realization was retained across jammer levels to preserve paired evaluation. Detection probability was computed using the unchanged source-calibrated threshold derived within the corresponding run, and ROC-AUC was evaluated from the corresponding clean and jammer-injected target scores.

The complete source-training, calibration, and target-evaluation procedure was repeated over five physically independent end-to-end runs. Across runs, the nominal source and target parameters were held fixed, whereas the packet realizations, desired- and interfering-channel coefficients, noise, co-channel interference, synchronization offsets, jammer realizations, and CAE initialization were independently regenerated. Within each run, one CAE and one OC-SVM were fitted and calibrated from the corresponding source-domain training and calibration partitions and were then transferred unchanged to all four target conditions. The CAE and OC-SVM were evaluated on the same physical realizations within each run. Run-level metrics for both detectors were summarized using the sample mean and two-sided 95% Student-*t* confidence interval,

(21) $$\bar{z}\pm t_{0.975,4}\frac{s_{z}}{\sqrt{5}},\;t_{0.975,4}=2.776.$$

where *z* denotes the corresponding run-level metric and $s_{z}$ its sample standard deviation across the five independent runs. The resulting intervals therefore quantify between-run variability arising from independent physical realizations and, for the CAE, model initialization and training, rather than training-seed variability on a fixed physical dataset. Although the OC-SVM is deterministic for a fixed dataset, its reported metrics vary across runs because each run contains independently generated physical training, calibration, and target-test realizations. For each target condition, the evaluation reports the realized clean-target false-alarm probability, the detection probability over the complete jammer-power sweep, and ROC-AUC. The corresponding results are presented in Section 6.7.

## 6. Results

### 6.1. Behavior of the Dechirp Statistic

Figure 2 shows the distribution of Π against relative jammer level for four representative conditions. Three distinct regimes are visible. Under co-channel interference and carrier-frequency offset, the nominal statistic remains high and tightly concentrated, with Π=171.6 at CCI=0dB and Π=164.3 at $\Delta f_{0}=+2000$ Hz. At the weakest jammer level tested, the statistic decreases by roughly a factor of two and the nominal and jammed distributions become almost disjoint. Under additive noise at SNR=0dB, the nominal value falls to 16.97 and the separation, although still pronounced, becomes narrower. Under severe Rayleigh fading, the nominal distribution is both lower (85.6) and substantially more dispersed, producing considerable overlap between nominal and jammed observations.

These representative conditions already anticipate the detector behavior observed below. For a concentration-based detector, sensitivity depends not only on the jammer-induced displacement of the statistic but also on the dispersion of the nominal reference against which that displacement is judged. Severe fading is particularly unfavorable in this respect: the attack shifts the statistic, but the nominal distribution is sufficiently broad that a fixed lower-tail threshold loses discriminative power.

### 6.2. Detection Characteristics Under Isolated Impairments

Figure 3 and Figure 4 report detection probability at $P_{\mathrm{FA}}=10^{-2}$ and threshold-independent AUC, respectively, for the four evaluated detector outputs.

The differences between Figure 3 and Figure 4 are central to the evaluation framework. Under co-channel interference at 0dB (panel c), the CAE detection probability is exactly zero from L=−10dB up to L=−3dB, only 0.003 at L=0dB, and 0.77 at the upper sweep limit. In contrast, PMR already achieves $P_{D}=0.70$ at L=−10dB and 0.97 at L=−8dB, with an AUC of 0.985 at the weakest jammer level, while the OC-SVM provides complete detection across the entire sweep. Thus, the strong co-channel condition does not eliminate attack-related information from the received preamble, even though the CAE reconstruction-error score fails to exploit it at weak jammer levels.

Under severe Rayleigh fading (panel b), the ranking reverses. The CAE reaches $P_{D}=0.25$ at L=−10dB, 0.95 at L=0dB, and 0.998 at L=+6dB. The OC-SVM trails the CAE and reaches $P_{D}=0.91$ only at L=+6dB, while PMR performs worst: its detection probability never exceeds 0.08 anywhere in the sweep, so the 0.9 sensitivity criterion is not attained. Under additive noise at SNR=0dB (panel a), PMR provides the strongest sensitivity, followed by the CAE and OC-SVM. Under the representative synchronization-impairment condition (panel d), all detectors reach high detection probabilities at weak jammer levels.

The AUC view in Figure 4 largely preserves these detector differences but substantially compresses their operational significance. Under co-channel interference at 0dB and L=0dB, for example, the CAE attains an AUC of 0.887 while its detection probability at the prescribed $P_{\mathrm{FA}}=10^{-2}$ operating point is only 0.003. Under severe fading at L=−10dB, the corresponding pair is 0.895 and 0.246. In both cases, substantial threshold-independent class separability coexists with poor detection at the selected low-false-alarm operating point. This divergence is precisely what the sensitivity floor of Equation (6) is designed to expose. The remainder of the analysis therefore emphasizes sensitivity floors and early-warning margins while retaining AUC as a complementary measure of separability.

### 6.3. Sensitivity Floors and Early-Warning Margins

Table 4 collects the detection sensitivity floors, preamble-based link-degradation onsets, and resulting early-warning margins. Figure 5 provides a graphical comparison of representative conditions, while the complete set of values is reported in Table 4.

Four observations follow. Table 5 reports, for the two learned detectors, the 95% bootstrap confidence interval of each uncensored floor and the range of the point estimate over five physically independent realizations of each condition. The bootstrap half-widths of the uncensored floors range from 0.25 to 1.3dB, whereas the realization-to-realization range reaches 4.1dB under severe fading and 1.9dB under co-channel interference at −3dB; under the synchronization impairments, the CAE floor is left-censored in the first realization but reaches −2.1dB in others. Where the PMR floor is resolved, its bootstrap half-width lies between 0.4 and 1.2dB, and the half-width of the degradation onset never exceeds 0.15dB. Detector differences smaller than these ranges are therefore not interpreted below.

First, and most consequentially, strong co-channel interference separates the detectors by more than 14dB of sensitivity. At CCI=0dB, the CAE does not reach the $P_{D}=0.9$ criterion anywhere within the evaluated sweep (its detection probability at L=+6dB is 0.77), whereas PMR reaches it at $L_{0.9}^{★}=-8.45\;\mathrm{dB}$ and the OC-SVM already satisfies the criterion at the lower sweep boundary, implying

$$L_{0.9}^{★}\leq -10\;\mathrm{dB}.$$

The sensitivity difference relative to the CAE therefore exceeds 14.45dB for PMR and 16dB for the OC-SVM. Since $L_{0.05}^{\mathrm{SER}}$ is common to all detectors under the same channel and attack condition, this result demonstrates that discriminative evidence of the jammer is already present in the received preamble at levels where the CAE reconstruction-error score fails to exploit it. The corresponding early-warning margin is not attained for the CAE, whereas it is +6.58dB for PMR and at least +8.13dB for the OC-SVM.

Second, early-warning margins can become negative. Under additive noise at SNR=0dB, the CAE and OC-SVM reach their sensitivity floors 0.90 and 3.28dB, respectively, beyond the preamble-degradation onset, corresponding to early-warning margins of −0.90dB and −3.28dB. Thus, under the selected operating criterion, neither learned detector provides a positive early-warning margin in this condition. PMR, by contrast, retains a +6.12dB margin. Table 5 shows that the corresponding CAE sensitivity-floor bootstrap interval is [+1.14,+1.74]dB, while the degradation-onset interval is [+0.49,+0.65]dB. Under severe fading, both learned detectors reach the criterion only after the degradation onset: the CAE margin is −0.22dB, with a bootstrap interval of [−1.15,+1.52]dB that includes zero, and the OC-SVM margin is −7.58dB, while PMR does not attain the criterion within the sweep. Under co-channel interference at −3dB, the CAE margin is likewise negative at −4.06dB, whereas PMR retains a positive margin of +8.16dB.

Third, the lowest-SNR condition exposes a regime in which weak-jammer discrimination is poor across the evaluated detector families. At SNR=−10dB and L=−10dB, the reported AUC values lie between 0.495 and 0.527, indicating essentially chance-level separability at the weakest jammer level. The common difficulty across detectors suggests that the received observations contain little usable evidence for weak-jammer discrimination under this operating condition, although the behavior at stronger jammer levels remains detector-dependent and PMR can exhibit the directional inversion described in Section 4.

Fourth, synchronization impairments are comparatively benign for detection under the evaluated configurations. Across nominal CFO values up to ±2 kHz, packet-to-packet frequency jitter, and sampling-time offsets of up to 64 samples, every reported early-warning margin exceeds 10dB, with most values left-censored and therefore known only as lower bounds. This result shows that when the synchronization variability is represented in the nominal detector distribution, these offsets produce substantially less loss of detection sensitivity than severe fading or strong co-channel interference. This holds for the first realization of each synchronization condition; across the five independent realizations, the CAE floor under CFO/STO ranges from below −10 to −2.1dB (Table 5), so the margin remains positive in every case, but its value is realization-dependent.

### 6.4. The Power-Domain Early-Warning Region

Figure 6 overlays jammer-induced preamble degradation on the detector sensitivity floors and makes the operational interpretation of the early-warning margin explicit.

Panel (a) shows the most pronounced case. Under co-channel interference at 0dB, the preamble-SER increment remains below 0.005 throughout the region in which PMR and the OC-SVM already achieve $P_{D}>0.96$. The PMR floor lies at −8.45dB and the OC-SVM floor is left-censored at −10dB, while the selected preamble-degradation onset occurs at −1.87dB. Consequently, the resulting power-domain early-warning margin is 6.58dB for PMR and at least 8.13dB for the OC-SVM. Within this region, the attack satisfies the monitoring criterion while the preamble remains below the selected degradation threshold. The CAE, by contrast, does not reach the detection criterion before the degradation onset, nor anywhere within the evaluated sweep.

Panel (b) illustrates the opposite behavior under severe fading. The nominal channel already introduces substantial preamble degradation, and the additional jammer-induced deterioration occurs at much lower relative jammer levels. The resulting power-domain early-warning margin is therefore compressed, and in this realization both learned detectors reach the detection criterion only after the selected onset. Panel (c) shows the corresponding noise-limited case at SNR=0dB, where PMR retains a positive early-warning margin while the two learned detectors cross their sensitivity criteria only after the selected degradation onset.

### 6.5. Complementarity and Fusion

The contrasting behavior of CAE and PMR across the isolated impairment families suggests that their scores contain partially non-redundant evidence. Figure 7 examines this relationship directly.

The median Spearman correlation between the standardized scores ranges from −0.195 to +0.571 depending on the channel condition. It remains close to zero under additive noise (0.014 at SNR=−10dB and 0.042 at 0dB) and under severe fading (−0.032), consistent with the substantially different detector responses observed in those regimes. The highest median dependence occurs under co-channel interference at 0dB, where the correlation reaches 0.571 and rises to 0.853 at the weakest jammer level. Even in this regime, however, similar score ordering does not imply equal sensitivity: PMR reaches the prescribed operating point more than 14dB below the CAE, which does not reach it within the evaluated sweep.

The weight-free maximum rule of Equation (18) was expected to exploit this complementarity. Under the independent calibration protocol, however, the Run-1 fused sensitivity floors in Table 4 coincide with the corresponding CAE floors across the fading, co-channel, and synchronization conditions. In particular, under co-channel interference at −3dB, both the CAE and the fused detector reach $L_{0.9}^{★}=+3.04\;\mathrm{dB}$. Although the point estimates coincide in this realization, their uncertainty differs: the fused floor has a 95% bootstrap interval of [−3.42,+3.76]dB and a realization range of [+0.39,+4.94]dB across the five physically independent runs. Under co-channel interference at 0dB, the fused detector does not attain $P_{D}=0.9$ within the evaluated sweep, whereas PMR alone reaches the criterion at −8.45dB. The limitation is condition-dependent: under additive noise at SNR=0dB, where the nominal PMR distribution is tightly concentrated, the fused floor of −5.30dB follows the PMR floor of −5.55dB rather than the CAE floor of +1.46dB.

The mechanism is visible in the standardized scores. The nominal PMR distribution is broad under interference, with an interquartile range of 60–75 on the raw scale, and the statistic is bounded, so the standardized jammed PMR score saturates between 2.4 and 4.6 even at the strongest jammer level. The standardized CAE score, in contrast, has a heavier nominal tail, with its 99th percentile ranging from 2.8 to 6.0, and its anomaly score can continue increasing under jamming. The 99th percentile of the fused nominal distribution is therefore governed by the heavier CAE tail, and the bounded PMR evidence rarely exceeds the resulting fused threshold. Consequently, the fusion rule preserves threshold-independent separability—under co-channel interference at 0dB and L=−10dB, the fused AUC is 0.917, compared with 0.567 for the CAE—but does not recover the fixed-operating-point sensitivity of the dechirp statistic. Recovering that sensitivity would require a fusion rule whose operating threshold is not dominated by the heavier-tailed constituent, for example, per-constituent calibration followed by a logical combination, which is left for future work.

### 6.6. Severity-Graded Composite Channels

The isolated conditions used above are diagnostic instruments rather than deployment models. The composite ladder therefore evaluates whether detector behavior established under individually controlled impairments transfers to conditions in which several mechanisms act simultaneously. Figure 8 and Table 6 report the available composite results.

The preamble-domain degradation reference required to compute $L_{0.05}^{\mathrm{SER}}$ is not available for this campaign, and early-warning margins are therefore not assigned to C1–C3. The composite analysis instead focuses on the sensitivity floors and separability of the two learned reference detectors for which the complete ladder results are available. Its role is consequently to test the transferability of isolated-condition sensitivity trends rather than to provide a four-detector early-warning comparison.

Detection sensitivity deteriorates monotonically along the severity ladder for both learned detectors, but the deterioration becomes substantially more pronounced at the adverse step. The OC-SVM floor shifts by 0.57dB between C1 and C2, from −2.33 to −1.76dB, and then fails to reach the $P_{D}=0.9$ criterion anywhere within the evaluated jammer sweep under C3. The CAE similarly moves from +2.59 to +2.66dB between C1 and C2 and likewise fails to attain $P_{D}=0.9$ within the evaluated jammer range under C3.

At the adverse step, the two learned detectors converge toward comparably poor performance. Their mean AUC values fall to 0.747 for CAE and 0.717 for OC-SVM. The CAE is therefore marginally superior in sweep-averaged separability, whereas neither detector reaches the $P_{D}=0.9$ sensitivity criterion within the evaluated range. Figure 9 provides the corresponding ROC curves and shows that the degradation at C3 is not explained solely by threshold placement.

Two conclusions follow from the composite campaign. First, detector rankings established under isolated impairments do not necessarily transfer to jointly impaired channels. Under isolated severe fading, the CAE holds a 7.36dB sensitivity advantage over the OC-SVM, whereas under C1 and C2 the OC-SVM leads by 4.92 and 4.42dB, respectively. Under C3, neither detector reaches the prescribed $P_{D}=0.9$ criterion within the evaluated sweep. The preferred detector therefore depends on the complete impairment environment rather than on the presence of a single nominal channel mechanism.

Second, performance under jointly impaired channels cannot be inferred reliably by inspecting the isolated impairment results independently. C3 combines lower SNR, stronger co-channel interference, larger CFO, synchronization variability, and severe Rayleigh multipath. Several of these components are individually well tolerated in the isolated campaigns, yet their joint occurrence produces a sharp loss of sensitivity and separability for both learned detectors. The composite ladder therefore demonstrates that isolated-impairment evaluations can substantially overstate robustness if their conclusions are transferred directly to jointly impaired operating conditions.

### 6.7. Calibration Robustness Under Controlled Single-Parameter Shifts

The controlled cross-condition experiment of Section 5.5 evaluates how a fixed detector and source-derived operating threshold respond when a single physical-layer parameter is changed while the remaining nominal channel conditions are preserved. Unlike the condition-matched experiments, no target-domain recalibration is performed. The resulting clean-target false-alarm probability therefore provides a direct measure of how strongly each individual physical change displaces the nominal operating point established at $P_{\mathrm{FA}}=10^{-2}$. Table 7 reports the clean-target false-alarm probability together with representative fixed-threshold detection probabilities and threshold-independent AUC values.

The four controlled shifts produce markedly different responses despite changing only one physical-layer parameter at a time. The most pronounced effect occurs for the SNR change from 0 to +8 dB. With all remaining source parameters and the detector thresholds held fixed, the clean-target false-alarm probability increases to 0.4617[0.4094,0.5140] for the CAE and 0.4229[0.3854,0.4604] for the OC-SVM. Consequently, the source-calibrated $P_{\mathrm{FA}}=10^{-2}$ operating point is no longer preserved. Although both detectors reach high detection probabilities as jammer power increases, these values must be interpreted in the context of the elevated clean-target false-alarm rate. At L=+6 dB, the corresponding AUC values of 0.9844 and 0.9898 show that strong-jammer score separability remains present; thus, the dominant effect of the SNR change is a substantial degradation of fixed-threshold calibration rather than a complete loss of jammer-related information.

The other three parameter changes are considerably less disruptive. Reducing CCI from −3 to −9 dB yields clean-target false-alarm probabilities of 0.0127[0.0047,0.0207] for the CAE and 0.0130[0.0047,0.0213] for the OC-SVM. Changing the nominal CFO from +1000 to +500 Hz produces values of 0.0091[0.0015,0.0167] and 0.0096[0.0043,0.0149], respectively, while replacing the severe Rayleigh profile with a Rician channel having K=6 dB yields 0.0138[0.0021,0.0255] and 0.0130[0.0040,0.0220]. These operating points remain substantially closer to the prescribed 0.01 false-alarm level than in the SNR-shift experiment.

The jammer sweeps further show that these more moderate parameter changes do not prevent the detectors from progressively recovering strong discrimination as relative jammer power increases. For the CCI, CFO, and fading-law shifts, the CAE reaches $P_{D}=0.9938$, 0.9929, and 0.9950, respectively, at L=+6 dB, while the corresponding OC-SVM values are 0.9794, 0.9791, and 0.9835. The associated AUC values are above 0.998 for both detectors in all three cases. At lower jammer powers, however, the detection probabilities remain substantially smaller, showing that preservation of the nominal operating point does not imply immunity to jammer-power limitations.

Taken together, these results show that the effect of a changing physical environment is strongly parameter-dependent. Selected variations in CCI level, nominal CFO, and fading law can be accommodated with only modest changes in the source-calibrated operating point, whereas the evaluated SNR shift causes a pronounced loss of false-alarm control. The experiment therefore supports a more nuanced interpretation than assuming either complete robustness or universal failure under changing channel conditions: some single-parameter changes have limited impact on fixed-threshold operation, while others can substantially degrade its reliability.

This observation provides quantitative motivation for adaptive or drift-aware calibration while remaining outside the scope of an implemented online detector. The present experiment deliberately keeps the detector models and source-derived thresholds fixed within each physical run and transfers them unchanged to the corresponding target conditions. Future adaptive implementations could use changes in nominal score statistics to determine when threshold recalibration is required, while the proposed power-domain framework would continue to provide the common $\left(P_{D},P_{\mathrm{FA}}\right)$ basis for evaluating the resulting detector.

## 7. Discussion

### 7.1. Representational Versus Informational Failure

The most transferable result of this study is methodological. When a learned detector loses sensitivity under a given impairment, two explanations must be distinguished: the received signal may no longer contain sufficient discriminative evidence of the attack, or the detector may fail to expose and exploit evidence that remains present. Conventional aggregate metrics cannot by themselves separate these possibilities. This distinction is particularly relevant under structured LoRa interference, where legitimate co-channel transmissions and LoRa-like jamming share waveform characteristics that can make detector behavior difficult to interpret.

The dechirp-domain statistic provides a direct diagnostic reference for this distinction. At CCI=0dB and L=−10dB, PMR achieves an AUC of 0.985 and $P_{D}=0.70$, whereas the CAE reconstruction-error detector exhibits an AUC of approximately 0.567 and essentially no detection at the prescribed operating point. The received preamble therefore contains highly discriminative attack-related information under precisely the condition in which the CAE loses sensitivity. In this operational sense, the failure is detector-dependent rather than signal-limited.

This result should not be interpreted as proof that the CAE latent code itself contains no jammer-related information. The present experiments evaluate the complete reconstruction-based detection pipeline through its reconstruction-error score and do not separately probe the information retained inside the latent representation. Accordingly, “representational failure” is used here in the practical sense that the learned reconstruction mechanism and its associated anomaly score fail to expose discriminative evidence that remains accessible through a physically matched transformation.

The underlying contrast is physically interpretable. The CAE is optimized to minimize reconstruction error over the complete three-channel preamble representation and must therefore model the substantial structured variability introduced by a strong co-channel LoRa interferer. A weak additional jammer can consequently produce only a small change in the resulting reconstruction error. Dechirping, in contrast, transforms the deterministic LoRa chirp structure into a spectral representation in which changes in concentration become directly observable. The more general lesson is that when a communication waveform admits a physically matched transformation, a low-dimensional statistic constructed after that transformation can reveal attack evidence that may be difficult to exploit from the original high-dimensional representation.

### 7.2. No Free Lunch Across Impairments

The complementary failure of PMR under severe fading is equally informative. Multipath modifies spectral concentration for reasons unrelated to jamming, broadening the nominal PMR distribution and reducing the separation produced by a weak attack. At L=−10dB, the nominal PMR distribution has a median of 174.72 and an interquartile range of 77.50 under co-channel interference at 0dB, compared with a median of 81.42 and an interquartile range of 44.67 under severe fading. The corresponding IQR-to-median ratios increase from 0.44 under co-channel interference to 0.55 under severe fading. At the same jammer level, the PMR median decreases by 56.0% under co-channel interference but by only 24.9% under severe fading. Expressed relative to the nominal interquartile range, the absolute displacement therefore decreases from 1.26 to 0.45. Using the condition-specific threshold obtained exclusively from the independent clean calibration partition, the fraction of held-out jammed packets crossing the lower-tail PMR threshold decreases from 0.695 under co-channel interference to 0.020 under severe fading.

The lower-tail PMR test therefore loses power not because its statistic becomes completely insensitive to the jammer, but because the nominal reference distribution becomes broad relative to the jammer-induced displacement. Despite the substantial nominal PMR dispersion under strong co-channel interference, the weak jammer produces a considerably larger displacement relative to that dispersion than under severe fading. This behavior is consistent with the PMR sensitivity floor of −8.45dB at CCI =0dB, whereas PMR does not attain $P_{D}=0.9$ anywhere within the evaluated sweep under severe fading. The CAE is better positioned in this condition because fading-induced variability is represented during nominal training and can be incorporated into its learned reconstruction behavior. The resulting reversal of detector ranking is central to the paper: neither a physically matched scalar statistic nor a learned reconstruction model is universally superior across impairment geometries.

The score-level analysis reinforces this interpretation. Under severe fading and several AWGN conditions, the CAE and PMR scores exhibit correlations close to zero, indicating that their anomaly evidence is only weakly redundant in those regimes. Under strong co-channel interference, the dependence becomes substantially larger, yet PMR still reaches the prescribed operating point more than 14dB below the CAE, which does not reach it within the evaluated sweep. Correlation and operational sensitivity therefore describe different properties of the detector outputs.

This observation has an immediate design implication. A single aggregate performance value obtained by pooling heterogeneous channel conditions can conceal substantial condition-dependent failures, because the resulting value depends on the composition of the evaluated channel mixture. Per-condition sensitivity floors and early-warning margins expose these weaknesses directly. The fusion results further show that complementary detector evidence does not automatically translate into a more robust fixed operating point: under the evaluated maximum rule, the fused threshold can suppress the sensitivity advantage of the more favorable constituent.

### 7.3. Implications for Deployment

The results suggest three practical implications for gateway-side physical-layer monitoring. These implications concern relative jammer-power sensitivity rather than response time: the reported margins quantify power-domain separation from the selected preamble-degradation onset and do not imply a corresponding temporal warning interval under arbitrary jammer-power dynamics. First, the dechirp-domain statistic is a strong candidate for continuous auxiliary monitoring whenever the receiver already exposes the dechirped frequency-domain preamble representation. Its additional processing consists primarily of peak and mean operations over quantities derived from the same dechirp/transform chain, while the evaluated interference- and synchronization-dominated conditions provide early-warning margins between 6.6 and 8.2dB under co-channel interference at 0 and −3dB and above 10dB under synchronization impairments, in several cases only as lower bounds because the sensitivity floor lies beyond the lower sweep limit.

Second, learned detectors remain valuable when propagation variability dominates. Severe Rayleigh fading provides the clearest example: PMR fails to attain $P_{D}=0.9$ anywhere in the evaluated jammer range, whereas the CAE reaches a sensitivity floor of −1.53dB, more than 7dB ahead of the OC-SVM; under the selected degradation criterion, its margin is −0.22dB, statistically indistinguishable from zero, but it remains the most sensitive detector in this regime. This complementary behavior motivates combining the learned and dechirp-domain paradigms, but the evaluated maximum rule does not achieve this at the prescribed operating point: under independent calibration, the fused floor follows the CAE floor across the fading, co-channel, and synchronization conditions of the first realization, because the bounded dechirp statistic can saturate below the fused threshold. Retaining PMR as a separate monitored decision is therefore the more reliable option for the evaluated configuration.

Third, the composite-channel results caution against extrapolating isolated-condition robustness to jointly impaired links. From C1 to C3, both learned detectors exhibit monotonic sensitivity degradation, with the adverse NLOS condition producing a sharp reduction in both fixed-operating-point detection and threshold-independent separability. Because a preamble-degradation reference was not available for the composite campaign, these results do not establish composite early-warning margins. They do show, however, that a detector that appears robust to several impairments individually can become substantially less effective when those mechanisms act simultaneously. In strongly impaired environments, physical-layer monitoring may therefore benefit from complementary evidence rather than reliance on a single detector or signal representation.

### 7.4. Threats to Validity

Several limitations bound the scope of these conclusions. First, this study is simulation-based. The waveform generation and impairment models follow established LoRa signal-processing and wireless-channel formulations, and the transmitter is based on the published GNU Radio implementation of Tapparel and Burg [21]; however, hardware-specific effects such as power-amplifier nonlinearity, oscillator phase noise, quantization, receiver front-end filtering, and automatic gain control are not explicitly represented. Such effects could modify the nominal and jammed score distributions and therefore alter the absolute sensitivity floors and early-warning margins observed here. Accordingly, the reported operating points should be interpreted as controlled simulation-domain estimates under the stated signal and channel models rather than as hardware-validated operating thresholds. Quantifying the magnitude and direction of hardware-induced changes would require future over-the-air validation under controlled offline experimental conditions, where the jammer-power sweep and the remaining physical-layer variables can be kept reproducible.

Second, the evaluation considers a single LoRa physical-layer configuration, SF=7 and B=125 kHz. Although the selected SF=7, B=125 kHz pair corresponds to a standardized LoRaWAN PHY configuration [44], the numerical detector sensitivities and pre-degradation margins reported here have only been evaluated for this configuration and should not be assumed to transfer unchanged to other LoRa PHY settings. Their dependence on other spreading factors, bandwidths, and adaptive-data-rate operating conditions remains outside the scope of the present study. The mathematical upper bound of the PMR statistic scales with the number of samples per symbol and therefore with the spreading-factor-dependent transform size. This does not imply, however, that higher spreading factors will necessarily improve detection sensitivity or early-warning margins; the interaction between spreading factor, propagation, interference, and nominal PMR dispersion remains outside the present evaluation.

Third, the jammer is represented by a single LoRa-like waveform injected at a fixed offset of 20 samples within the first preamble symbol. Reactive strategies with different triggering delays, frequency-selective jammers, multiple simultaneous adversaries, and protocol-aware attacks are therefore outside the present threat model. Likewise, an adversary deliberately designed to preserve dechirp-domain spectral concentration could specifically reduce the effectiveness of PMR. The proposed statistic should consequently be interpreted as a detector for the evaluated preamble-directed LoRa-like jammer model rather than as a universal jamming detector. Evaluating variable-delay reactive attacks, frequency-selective strategies, and adaptive or protocol-aware adversaries remains an important future extension for determining how the proposed power-domain sensitivity framework behaves under a broader threat space.

Fourth, the severity-graded composite ladder contains only three controlled operating points. Its purpose is to test transferability under progressively more adverse joint impairments, not to statistically represent the complete distribution of deployed LoRa channels. The monotonic degradation observed from C1 through C3 is therefore an empirical result for this controlled ladder and should not be extrapolated beyond the evaluated configurations. In addition, the complete composite campaign is available only for CAE and OC-SVM, and the preamble-domain reference required to compute $L_{0.05}^{\mathrm{SER}}$ and $M_{0.9}$ was not retained. Consequently, the composite results establish sensitivity degradation and ranking changes for the learned detectors, but not composite early-warning margins or fusion performance.

Fifth, the detector-agnostic character of the proposed framework refers to its applicability across different monitoring algorithms and should not be interpreted as implying adaptive or online operation of the detector implementations evaluated here. The condition-matched experiments use fixed model and score-calibration procedures associated with each controlled nominal regime, whereas the complementary cross-condition campaign of Section 5.5 and Section 6.7 evaluates how fixed learned detectors and source-derived thresholds respond when a single physical-layer parameter is changed while the remaining nominal conditions are preserved. No target-domain recalibration, online model updating, or temporal state adaptation is performed. The results show that some controlled changes, such as those evaluated for CCI, nominal CFO, and fading law, preserve the source-calibrated operating point relatively closely, whereas the evaluated SNR change produces a substantially larger false-alarm displacement. Consequently, this campaign should be interpreted as a controlled calibration-robustness stress test rather than as an implementation of an adaptive or self-calibrating monitor. Adaptive threshold tracking and online monitoring under evolving nominal statistics therefore remain important extensions of the proposed framework.

Sixth, the PMR and preamble-SER calculations reported for the CFO/STO conditions use a reference synchronization convention in which the true sampling-time offset is used for preamble alignment and deterministic FFT-bin compensation is applied for the carrier-frequency offset, without time-domain CFO correction. This convention was retained to evaluate the dechirp-domain statistic under controlled synchronization impairments and should not be interpreted as a practical estimated synchronization front end. The CAE and OC-SVM inputs do not use this reference PMR alignment. Consequently, the synchronization-related PMR and preamble-SER results should be interpreted as a controlled reference case under known synchronization offsets; practical implementations based on estimated timing and frequency offsets may exhibit additional sensitivity loss. Evaluating the dechirp-domain detector with fully estimated synchronization therefore remains an important extension for future work.

Finally, the detector-evaluation protocol separates jamming-free training, validation, calibration, and held-out test populations, as detailed in Section 5.4. Operating thresholds are estimated exclusively from the clean calibration partition, while realized false-alarm performance is evaluated on an independent held-out clean test partition. Jammer-injected test observations are generated only from the frozen test population after all model-development and threshold-calibration operations have been completed. This separation limits information leakage between detector development and performance evaluation; however, the reported results remain conditioned on the simulated signal, channel, and threat models considered in this study.

## 8. Conclusions

This work argues that physical-layer jamming monitors for LoRa should be evaluated not only by whether an attack is eventually detectable, but by how much jammer-power separation exists between reliable detection and the selected preamble-degradation onset. To make this relationship quantitative, we introduced three quantities defined on a common relative jammer-power axis: the detection sensitivity floor, the preamble-degradation onset, and the power-domain early-warning margin separating them. The resulting framework expresses pre-degradation detection capability directly in decibels and enables heterogeneous monitoring strategies to be compared at the same prescribed $\left(P_{D},P_{\mathrm{FA}}\right)$ operating point. The margin therefore represents jammer-power headroom rather than elapsed warning time and does not, by itself, imply a specific temporal opportunity for response under rapidly changing jammer power.

Applied to 221 isolated channel–jammer operating points and complemented by a three-step severity-graded composite ladder, the framework exposes behavior that conventional aggregate metrics can obscure. Under SNR=0dB and under severe fading, the point-estimate CAE and OC-SVM power-domain early-warning margins are negative, reaching the required detection probability only after the selected preamble-degradation onset. Under strong co-channel interference, by contrast, the dechirp-domain PMR statistic reaches the $P_{D}=0.9$ criterion at −8.45dB, whereas the CAE does not reach it anywhere within the evaluated sweep. The resulting sensitivity advantage therefore exceeds 14.45dB, demonstrating that the pronounced CAE degradation in this regime is detector-dependent rather than evidence that discriminative jammer information has disappeared from the received preamble.

No detector is universally dominant. PMR is particularly effective under strong co-channel interference and synchronization impairments, but becomes the weakest detector under severe Rayleigh fading because propagation-induced spectral dispersion broadens its nominal reference distribution. The CAE exhibits the opposite advantage in this regime, reaching the required detection probability 7.36dB before the OC-SVM, while PMR does not attain it at all. These contrasting failure modes are reflected in the score-level analysis and motivated the evaluated fusion rule. Under independent threshold calibration, however, the weight-free maximum rule does not recover the fixed-operating-point sensitivity of PMR: across the fading, co-channel, and synchronization conditions of the first realization, its sensitivity floor follows the CAE floor, while the bounded dechirp statistic can saturate below the fused threshold set by the heavier-tailed reconstruction-error score. The pre-degradation advantage of the dechirp statistic is therefore more reliably retained by monitoring it as a separate decision than through the evaluated maximum rule.

The composite-channel campaign provides a complementary result. Although early-warning margins cannot be computed for C1–C3 because the corresponding preamble-degradation reference was not retained, detection sensitivity deteriorates monotonically along the severity ladder for both learned detectors. Across the four physically independent runs, the mean OC-SVM sensitivity floor shifts from −2.33dB in C1 to −1.76dB in C2, while the CAE shifts from +2.59dB to +2.66dB. Under the adverse C3 condition, neither detector attains $P_{D}=0.9$ within the evaluated jammer range in any of the four runs. Threshold-independent separability also deteriorates sharply at C3, where the sweep-averaged mean AUC decreases to 0.747 for the CAE and 0.717 for the OC-SVM. Moreover, detector rankings observed under isolated impairments do not necessarily transfer to jointly impaired channels. These results show that robustness inferred from one-impairment-at-a-time evaluation can substantially overstate performance under composite physical-layer conditions.

The additional controlled cross-condition campaign further shows that the robustness of a fixed source-calibrated operating point depends on the physical parameter that changes. Across five physically independent runs, controlled changes in CCI level, nominal CFO, and fading law preserved the prescribed false-alarm operating point relatively closely, whereas increasing the SNR from 0 to +8 dB produced a pronounced false-alarm increase, reaching a mean clean-target FPR of 0.4617 for the CAE and 0.4229 for the OC-SVM. At L=+6 dB, both detectors nevertheless retained strong threshold-independent separability under the SNR shift, with AUC values of 0.9844 and 0.9898, respectively, indicating that the dominant effect was a displacement of the fixed operating threshold rather than a complete loss of jammer-related score information. These results show that some nominal-channel variations can be accommodated with limited calibration change, while others can substantially degrade fixed-threshold operating reliability, thereby motivating adaptive or drift-aware calibration for deployment under evolving receiver conditions.

Future work should validate the proposed margins using over-the-air captures and systematically examine their dependence on spreading factors, bandwidth, adaptive-data-rate operation, and representative network-traffic and collision conditions. In particular, the detection sensitivity floor, preamble-degradation onset, and power-domain early-warning margin should be recomputed across multiple standardized LoRa PHY configurations and representative ADR transitions to quantify directly how changes in the underlying waveform affect detector calibration and pre-degradation warning capability. Future studies should also evaluate adversaries that deliberately modify their waveform or timing to preserve dechirp-domain spectral concentration. Additionally, building on the static score-level fusion examined in this study, future work should investigate adaptive threshold tracking, temporal aggregation of successive detector scores, and online fusion of complementary detector types under time-varying channel and interference conditions. These mechanisms may improve robustness to evolving nominal statistics while retaining the proposed power-domain sensitivity framework as a common basis for performance evaluation. More broadly, the early-warning formulation is not intrinsically tied to LoRa: communication systems with deterministic acquisition structures can, in principle, define an analogous separation between reliable attack detection and a measurable communication-side degradation onset. The central methodological message is therefore broader than the particular detectors considered here: detection performance becomes operationally more informative when it is measured relative to the degradation threshold of the signal structure that the monitor is intended to protect. 

## Figures and Tables

**Figure 1:**
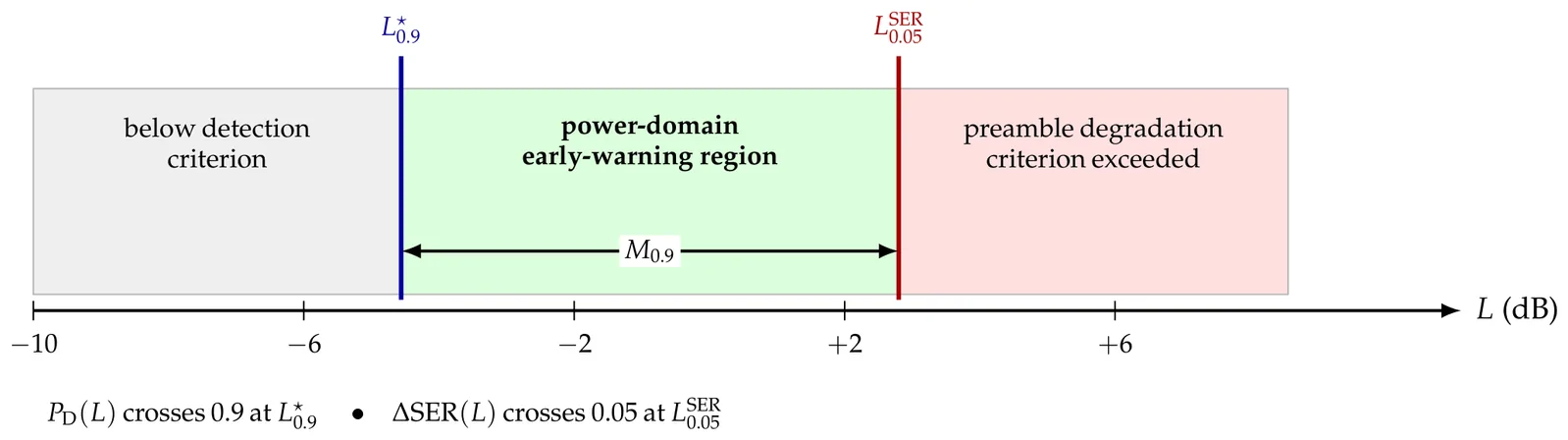
Definition of the three figures of merit on the relative jammer-level axis. The detection sensitivity floor $L_{0.9}^{★}$ and the preamble-based link-degradation onset $L_{0.05}^{\mathrm{SER}}$ are estimated independently from the same jammer-power sweep. Their separation defines the power-domain early-warning margin $M_{0.9}$, expressed in decibels of relative jammer power rather than as elapsed time. The framework is detector-agnostic: any monitoring method that produces a packet-level score and corresponding $P_{D}\left(L\right)$ characteristic can be evaluated on the same axis.

**Figure 2:**
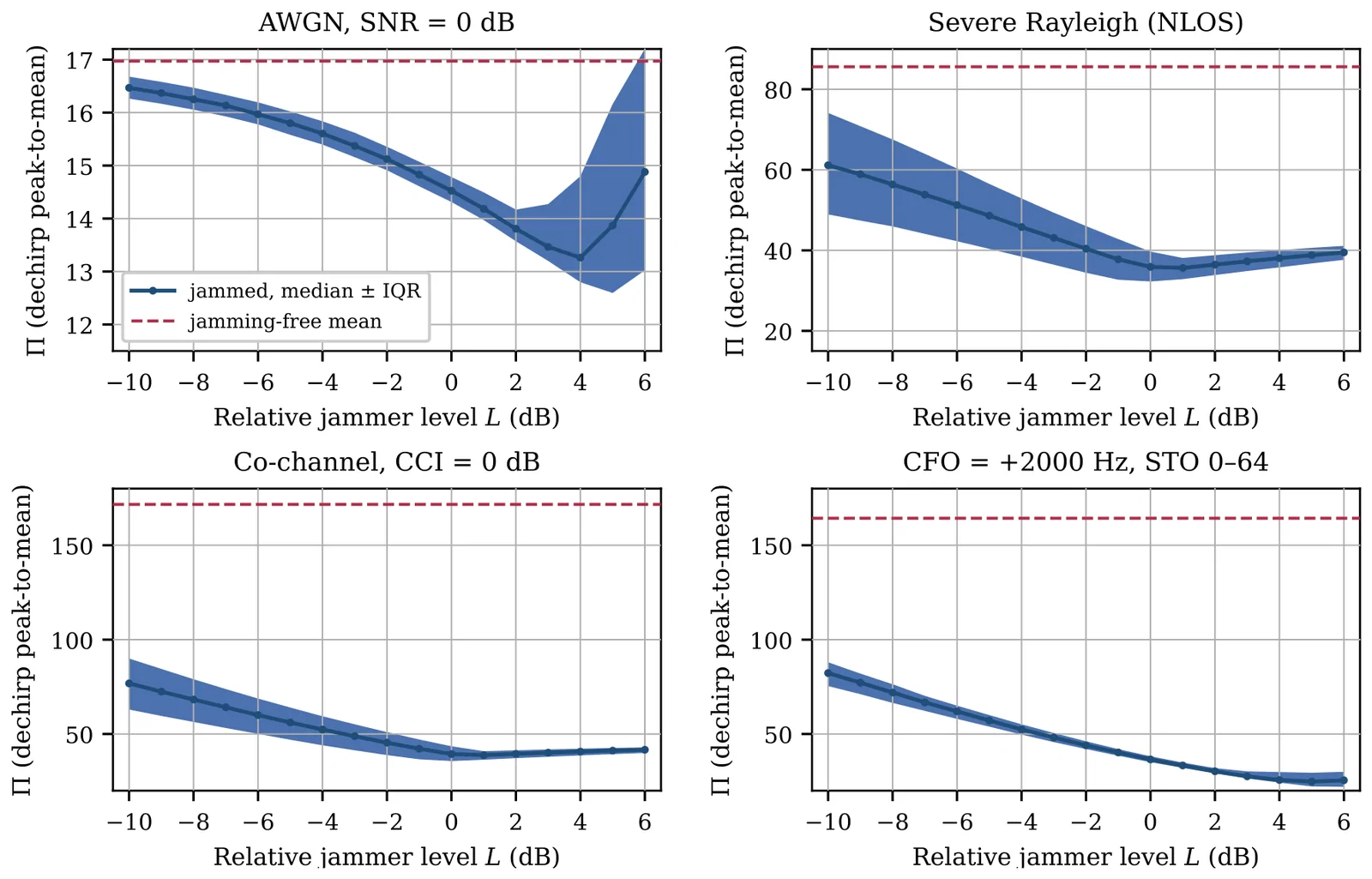
Dechirp-domain peak-to-mean statistic Π against relative jammer level for four representative conditions. Shaded bands span the interquartile range of the jammed population; the dashed line is the jamming-free mean. The statistic is bounded above by N=256. Note that the vertical scale differs across panels to preserve visibility of the condition-dependent PMR dispersion.

**Figure 3:**
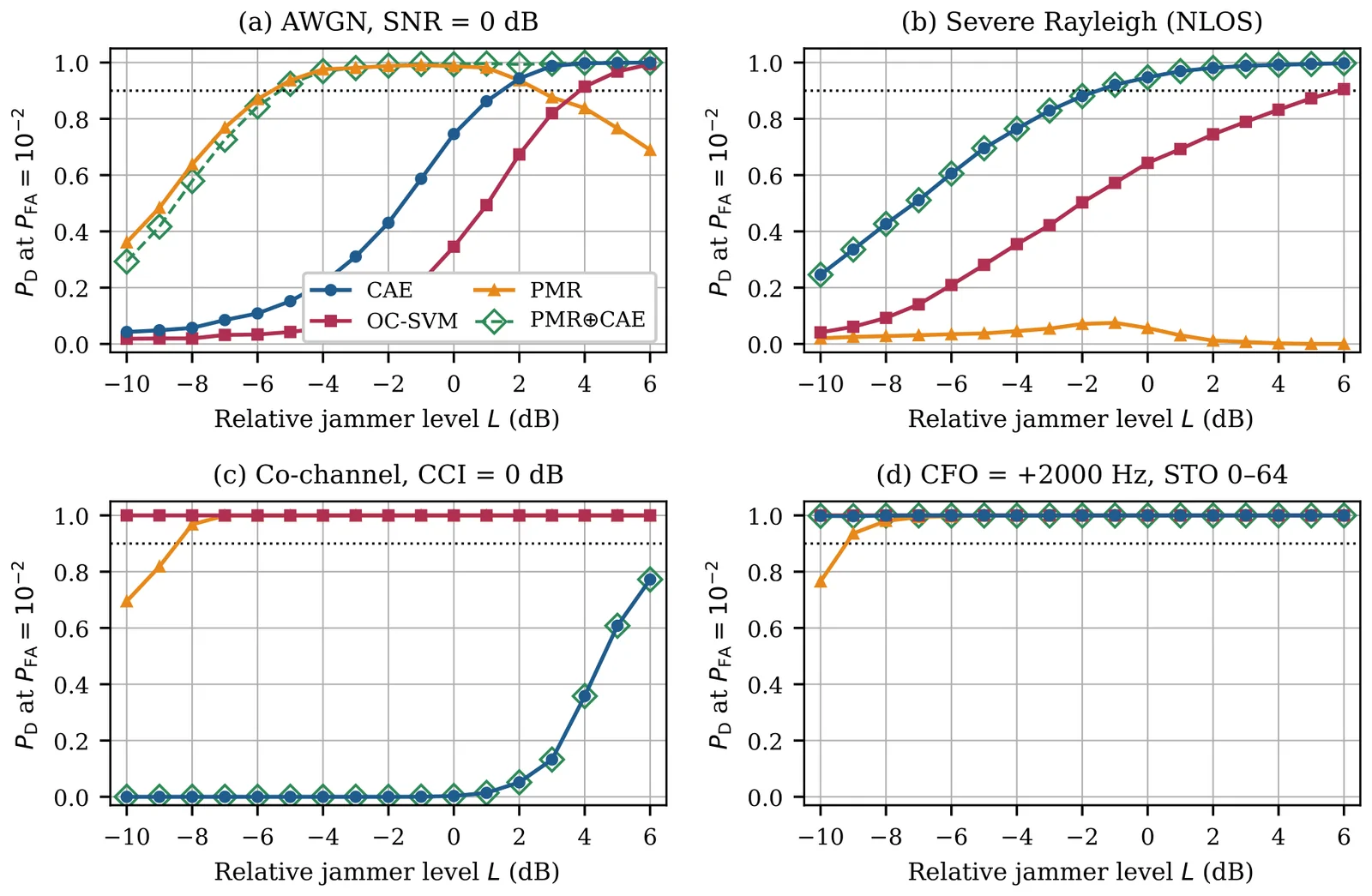
Detection probability at a fixed false-alarm probability of $10^{-2}$ against relative jammer level. The dotted line marks the $P_{D}=0.9$ criterion used to define the sensitivity floor.

**Figure 4:**
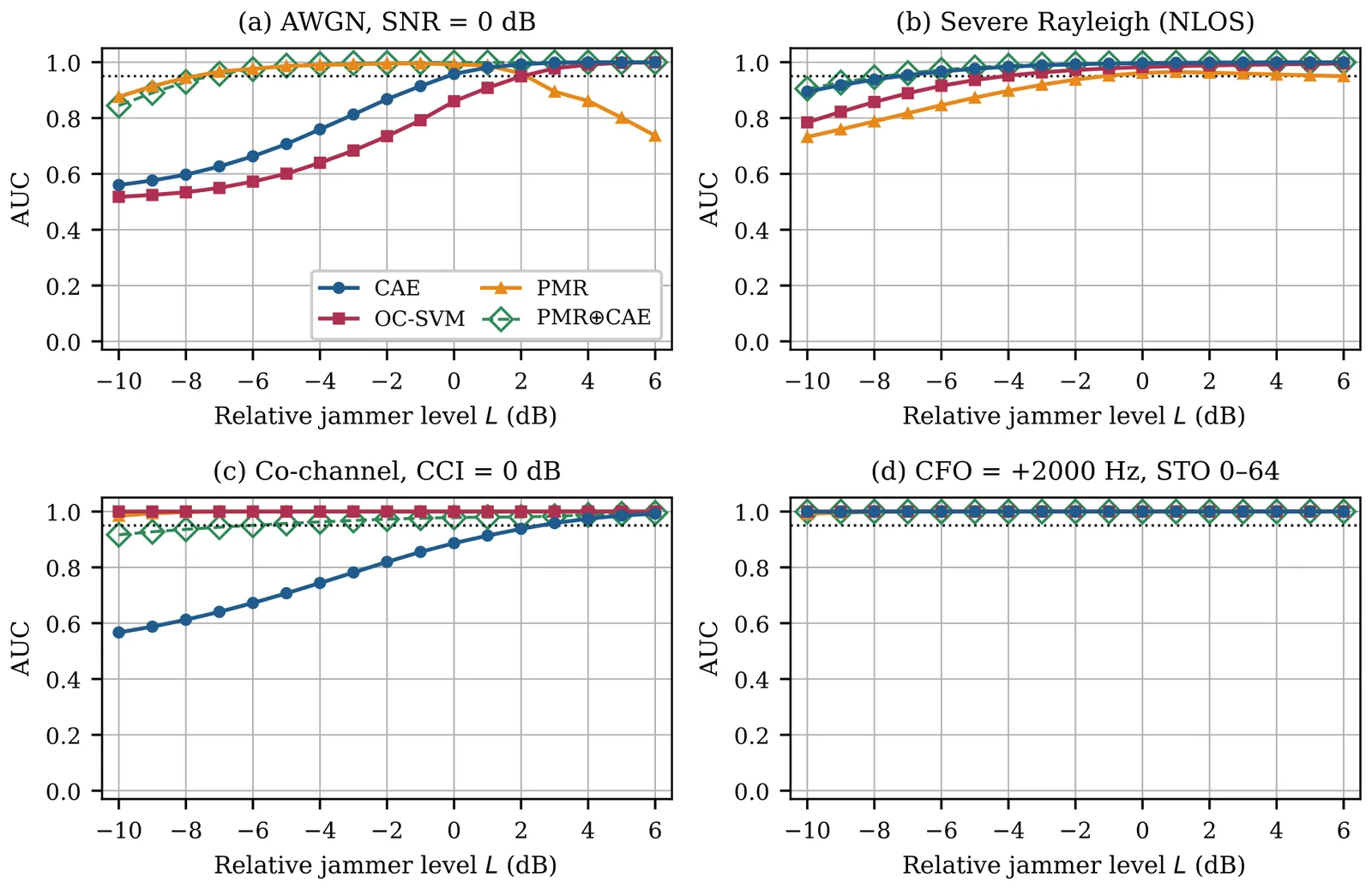
Threshold-independent separability, measured by the area under the receiver operating characteristic curve, for the same representative conditions as Figure 3.

**Figure 5:**
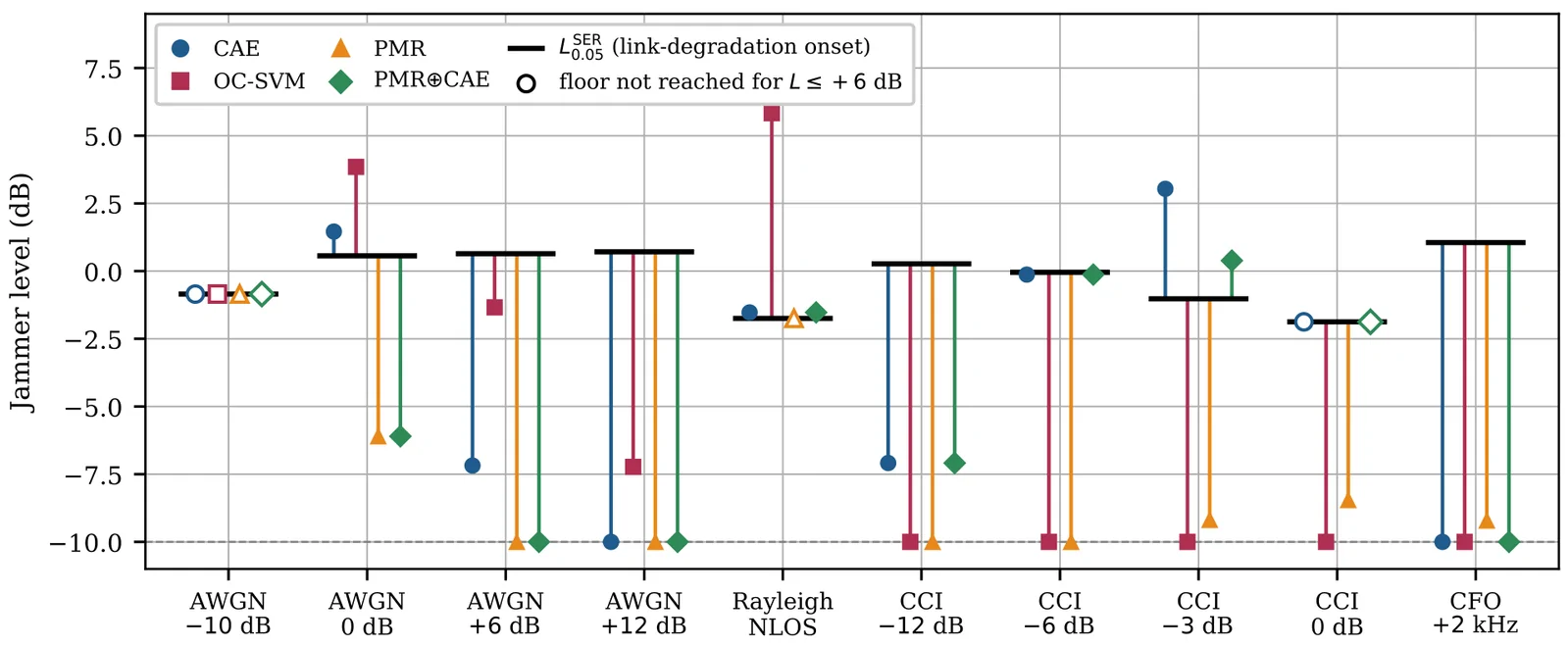
Sensitivity floors against the preamble-based link-degradation onset. The vertical connector represents the early-warning margin; connectors extending beyond the degradation reference indicate negative margins. Open markers denote conditions in which $P_{D}=0.9$ was not attained within the evaluated sweep. Points located at the −10 dB boundary should be interpreted as left-censored according to Table 4.

**Figure 6:**
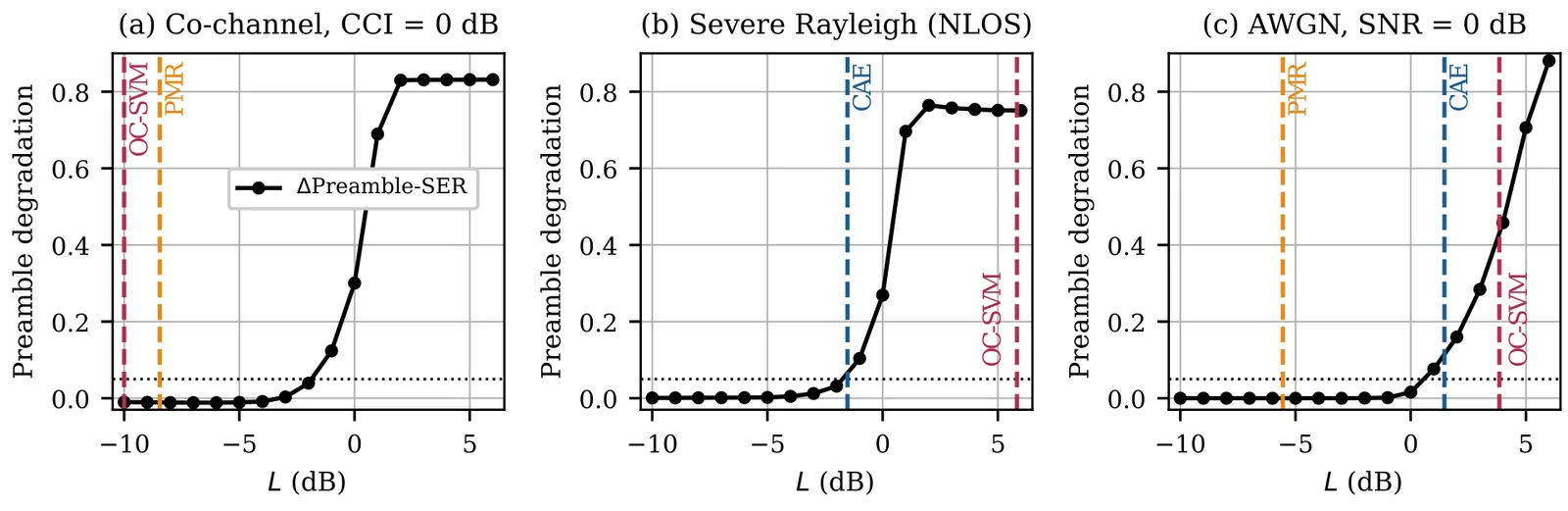
Preamble-SER degradation relative to the jamming-free condition, quantified as $\Delta \mathrm{SER}\left(L\right)=\mathrm{SER}\left(L\right)-{\mathrm{SER}}_{0}$, with detector sensitivity floors marked as vertical dashed lines. The interval between a detector sensitivity floor and the crossing of the dotted ΔSER=0.05 criterion defines its power-domain early-warning margin.

**Figure 7:**
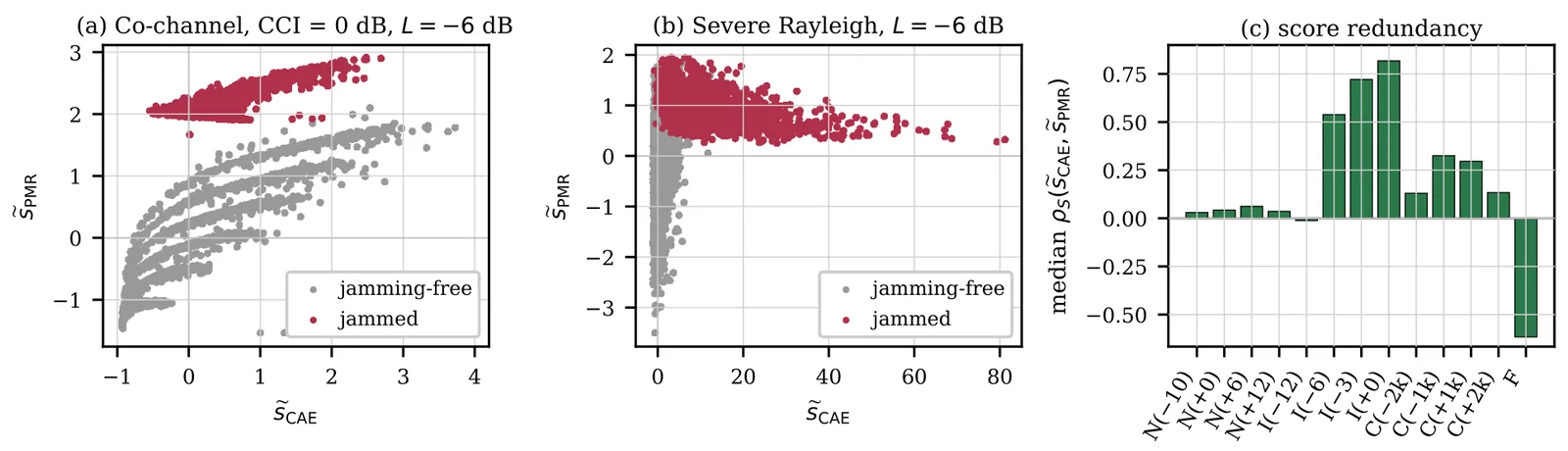
Complementarity of the two anomaly scores. Panels (**a**,**b**) show robustly standardized scores for nominal and jammed packets at L=−6 dB under co-channel interference and severe fading. Panel (**c**) reports the median Spearman correlation between the two standardized scores across the jammer sweep for every isolated condition, using the codes N (AWGN), F (severe fading), I (co-channel), and C (CFO).

**Figure 8:**
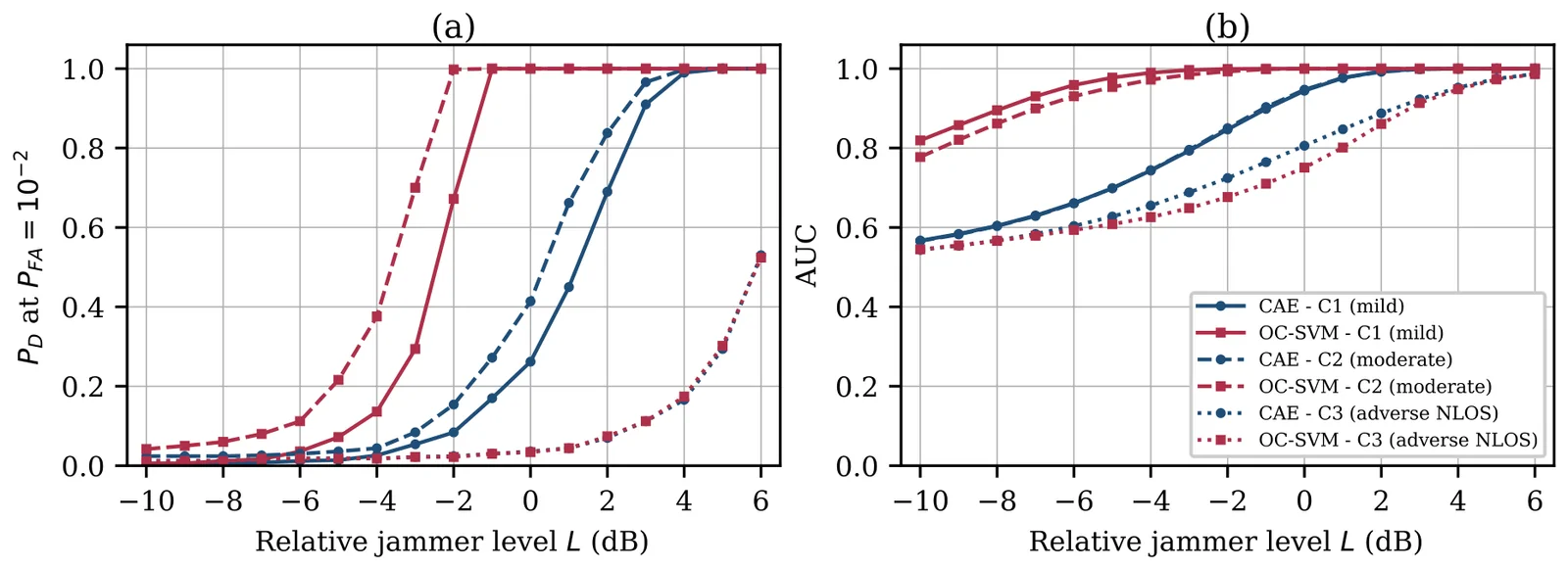
Detection probability (**a**) and area under the receiver operating characteristic curve (**b**) across the severity-graded composite ladder. Line style encodes severity, while marker and color identify the detector. The curves correspond to the first independent physical realization and are shown for visual comparison; between-run uncertainty is quantified separately in Table 6.

**Figure 9:**
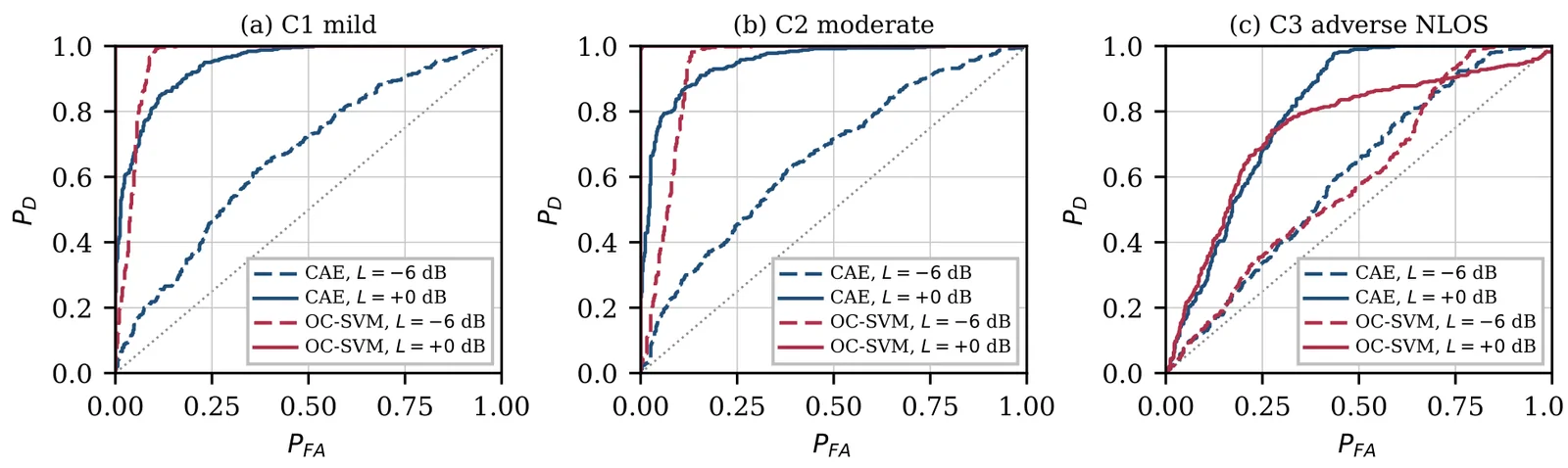
Receiver operating characteristics under the composite ladder at two jammer levels. The near-diagonal C3 curves at L=−6 dB show that statistical separability itself is strongly degraded at the adverse step, rather than the observed loss being attributable only to the selected operating threshold. The curves correspond to the first independent physical realization.

**Table 1:** Isolated impairment conditions. Each listed configuration is evaluated over the complete 17-point relative jammer-level sweep.

Family	Configuration	Levels Analyzed	Packets/Level
AWGN	$h_{s}=\delta$, $\alpha_{c}=0$, Δf=0	{−10,0,+6,+12} dB SNR	2000
Severe fading	Rayleigh, 5 taps at {0,1.5,3.5,7,12} μs with gains {0,−2.5,−5.5,−9,−13} dB, packet-wise block fading with nominal Doppler parameter 4 Hz and no intra-packet channel evolution; no additional AWGN	single profile	2000
Co-channel	Ideal desired branch and independent packet-constant flat-Rayleigh interferer; relative delay of 0–6 symbols on symbol boundaries (0,256,…,1536 samples). CCI is expressed as I/S in dB, with $\alpha_{c}=10^{{\mathrm{CCI}}_{\mathrm{dB}}/20}$; negative values denote an interferer weaker than the desired signal	{−12,−6,−3,0} dB	2000
CFO/STO	$\Delta f=\Delta f_{0}+\epsilon$, $\epsilon ∼N(0,50^{2})$ Hz truncated to ±150 Hz, STO ∼U{0,…,64} samples	{−2000,−1000,+1000,+2000} Hz	2000

**Table 2:** Severity-graded composite conditions. Each condition is evaluated over four independent runs. Within each run, 500 held-out clean test realizations and 500 corresponding jammer-injected test observations at each relative jammer level are used for evaluation, yielding an aggregate held-out population of 2000 observations per class and jammer level across the four runs. Statistical aggregation is performed at the run level rather than by treating the pooled packet-level observations as independent experimental replicates.

Parameter	C1 (Mild)	C2 (Moderate)	C3 (Adverse NLOS)
SNR	+10 dB	+10 dB	+6 dB
Co-channel level	−12 dB	−9 dB	−6 dB
Interferer delay	0–6 symbols, on symbol boundaries	as C1	as C1
Nominal CFO	+500 Hz	+500 Hz	+1000 Hz
CFO jitter	$\sigma_{\epsilon }=50$ Hz, truncated to ±150 Hz	$\sigma_{\epsilon }=50$ Hz, truncated to ±150 Hz	$\sigma_{\epsilon }=50$ Hz, truncated to ±150 Hz
STO	U{0,…,64}	U{0,…,64}	U{0,…,64}
Desired channel	Rician, K=12 dB, 3 taps {0,0.7,1.8}μs, gains {0,−4,−9} dB, packet-wise block fading (nominal Doppler parameter 0.8 Hz; no intra-packet channel evolution)	as C1	Rayleigh, 5 taps {0,1.5,3.5,7,12}μs, gains {0,−2.5,−5.5,−9,−13} dB, packet-wise block fading (nominal Doppler parameter 4 Hz; no intra-packet channel evolution)
Interferer channel	Independent packet-constant flat-Rayleigh fading	as C1	as C1

**Table 3:** Independent dataset architecture used in the detector evaluation. All quantities refer to jamming-free realizations before jammer injection, except for the jammed-test column.

Scenario	Train Clean	Validation Clean	Calibration Clean	Test Clean	Jammed Test/Level	Threshold Source	Test Isolation
AWGN, per SNR	1600	400	500	2000	2000	Calibration clean only	Before jammer injection
Severe fading	1600	400	500	2000	2000	Calibration clean only	Before jammer injection
CCI, per level	1600	400	500	2000	2000	Calibration clean only	Before jammer injection
CFO/STO, per nominal CFO	1600	400	500	2000	2000	Calibration clean only	Before jammer injection
Composite, per run	800	200	500	500	500	Calibration clean only	Before jammer injection

**Table 4:** Detection sensitivity floors $L_{0.9}^{★}$, preamble-based link-degradation onset $L_{0.05}^{\mathrm{SER}}$, and early-warning margins $M_{0.9}$, in decibels. Entries $L_{0.9}^{★}\leq -10.00$ are left-censored at the lower sweep boundary; the corresponding margins are therefore lower bounds. The notation n.a. indicates that $P_{D}=0.9$ was not attained for L≤+6 dB. All point estimates correspond to the first independent realization of the corrected disjoint evaluation campaign. Bootstrap confidence intervals and realization ranges across five physically independent realizations are reported in Table 5.

Condition	Level	$L_{0.9}^{★}$				$L_{0.05}^{\mathrm{SER}}$	$M_{0.9}$			
		CAE	OC-SVM	PMR	Fused		CAE	OC-SVM	PMR	Fused
AWGN	−10 dB	n.a.	n.a.	n.a.	n.a.	−0.85	n.a.	n.a.	n.a.	n.a.
AWGN	0 dB	+1.46	+3.85	−5.55	−5.30	+0.56	−0.90	−3.28	+6.12	+5.87
AWGN	+6 dB	−7.18	−1.34	≤−10.00	≤−10.00	+0.64	+7.82	+1.98	≥+10.64	≥+10.64
AWGN	+12 dB	≤−10.00	−7.23	≤−10.00	≤−10.00	+0.71	≥+10.71	+7.94	≥+10.71	≥+10.71
Rayleigh	severe	−1.53	+5.83	n.a.	−1.53	−1.75	−0.22	−7.58	n.a.	−0.22
Co-channel	−12 dB	−7.09	≤−10.00	≤−10.00	−7.09	+0.27	+7.35	≥+10.27	≥+10.27	+7.35
Co-channel	−6 dB	−0.13	≤−10.00	≤−10.00	−0.13	−0.04	+0.08	≥+9.96	≥+9.96	+0.08
Co-channel	−3 dB	+3.04	≤−10.00	−9.19	+3.04	−1.02	−4.06	≥+8.98	+8.16	−4.06
Co-channel	0 dB	n.a.	≤−10.00	−8.45	n.a.	−1.87	n.a.	≥+8.13	+6.58	n.a.
CFO/STO	−2000 Hz	≤−10.00	≤−10.00	−9.19	≤−10.00	+1.21	≥+11.21	≥+11.21	+10.40	≥+11.21
CFO/STO	−1000 Hz	≤−10.00	≤−10.00	≤−10.00	≤−10.00	+1.06	≥+11.06	≥+11.06	≥+11.06	≥+11.06
CFO/STO	+1000 Hz	≤−10.00	≤−10.00	≤−10.00	≤−10.00	+1.15	≥+11.15	≥+11.15	≥+11.15	≥+11.15
CFO/STO	+2000 Hz	≤−10.00	≤−10.00	−9.21	≤−10.00	+1.05	≥+11.05	≥+11.05	+10.27	≥+11.05

**Table 5:** Statistical uncertainty of the sensitivity floors $L_{0.9}^{★}$ and of the degradation onset $L_{0.05}^{\mathrm{SER}}$, in decibels. Brackets give 95% percentile bootstrap confidence intervals obtained from 2000 paired packet-level replicates of the first realization (Section 5.4); the range columns give the minimum and maximum of the point estimate over five physically independent realizations of each condition. Censored entries are reported without an interval when more than 95% of the replicates were censored in the same direction. PMR and $L_{0.05}^{\mathrm{SER}}$ entries are available for all isolated impairment conditions. Fusion uncertainty is not tabulated in full for compactness. For co-channel interference at −3dB, the fused floor has a 95% bootstrap interval of [−3.42,+3.76]dB and a realization range of [+0.39,+4.94]dB across the five physically independent runs.

Condition	Level	CAE		OC-SVM		PMR		$L_{0.05}^{\mathrm{SER}}$
		95% CI	Range (N=5)	95% CI	Range (N=5)	95% CI	Range (N=5)	95% CI
AWGN	−10 dB	n.a.	n.a.	n.a.	n.a.	n.a.	n.a.	[−0.94,−0.76]
AWGN	0 dB	[+1.14,+1.74]	[+1.46,+1.94]	[+3.55,+4.27]	[+3.85,+4.54]	[−6.01,−5.06]	[−5.55,−4.88]	[+0.49,+0.65]
AWGN	+6 dB	[−7.52,−7.02]	[−7.18,−6.92]	[−1.56,−0.97]	[−1.34,−0.85]	≤−10.00	≤−10.00	[+0.57,+0.74]
AWGN	+12 dB	≤−10.00	≤−10.00	[−7.44,−6.73]	[−7.23,−6.98]	≤−10.00	≤−10.00	[+0.63,+0.82]
Rayleigh	severe	[−3.29,−0.62]	[−5.65,−1.52]	[+3.57,n.a.]	[+4.02,n.a.]	n.a.	n.a.	[−1.85,−1.64]
Co-channel	−12 dB	[−8.48,−6.07]	[−7.64,−6.89]	≤−10.00	≤−10.00	≤−10.00	≤−10.00	[+0.25,+0.29]
Co-channel	−6 dB	[−1.12,+0.75]	[−1.65,−0.13]	≤−10.00	≤−10.00	[≤ −10.00,−9.63]	≤−10.00	[−0.21,+0.01]
Co-channel	−3 dB	[+1.90,+3.76]	[+3.04,+4.94]	≤−10.00	≤−10.00	[−9.84,−7.53]	[−9.90,−8.75]	[−1.22,−0.94]
Co-channel	0 dB	n.a.	n.a.	≤−10.00	≤−10.00	[−9.02,−8.28]	[−8.50,−7.91]	[−1.97,−1.78]
CFO/STO	−2000 Hz	≤−10.00	[≤ −10.00,−9.97]	≤−10.00	≤−10.00	[≤ −10.00,−9.02]	[−9.88,−8.41]	[+1.10,+1.31]
CFO/STO	−1000 Hz	≤−10.00	[≤ −10.00,−2.08]	≤−10.00	≤−10.00	[≤ −10.00,−9.12]	[≤ −10.00,−9.46]	[+0.94,+1.17]
CFO/STO	+1000 Hz	≤−10.00	[≤ −10.00,−2.14]	≤−10.00	≤−10.00	[≤ −10.00,−9.84]	≤−10.00	[+1.03,+1.24]
CFO/STO	+2000 Hz	≤−10.00	[≤ −10.00,−2.76]	≤−10.00	≤−10.00	[≤ −10.00,−8.26]	[−9.54,−9.21]	[+0.91,+1.17]

**Table 6:** Composite-ladder sensitivity and separability for the learned reference detectors. Unless censoring is indicated, each entry reports the mean across four physically independent runs followed by the run-level 95% Student-*t* confidence interval in brackets (N=4). In every run, thresholds were obtained exclusively from 500 clean calibration realizations and metrics from 500 held-out clean and 500 paired jammer-injected test realizations per jammer level. The sensitivity floors $L_{0.9}^{★}$ and $L_{0.5}^{★}$ were obtained by first-crossing linear interpolation on the 1-dB grid. The sweep-averaged AUC is the arithmetic mean over L∈[−10,+6] dB. The notation n.a. indicates that the target detection probability was not reached in any run. For mixed right-censoring, the bracket gives the range of the finite run estimates, followed by the number of finite and right-censored runs; a parametric confidence interval is not reported for such cells. Figure 8 displays the first independent run for visual clarity, whereas this table quantifies between-run uncertainty.

Condition	Detector	$L_{0.9}^{★}$ (dB)	$L_{0.5}^{★}$ (dB)	AUC at L=−10 dB	AUC at L=−0 dB	Mean AUC
		+2.59	+0.64	0.569	0.947	0.822
C1 (mild)	CAE	[+2.19,+2.99]	[+0.03,+1.25]	[0.563,0.574]	[0.936,0.957]	[0.814,0.831]
		−2.33	−3.33	0.821	1.000	0.966
C1 (mild)	OC-SVM	[−3.54,−1.13]	[−4.34,−2.33]	[0.805,0.837]	[0.999,1.000]	[0.960,0.971]
		+2.66	+0.70	0.567	0.944	0.820
C2 (moderate)	CAE	[+2.09,+3.22]	[−0.16,+1.57]	[0.563,0.571]	[0.934,0.954]	[0.814,0.827]
		−1.76	−2.95	0.773	0.999	0.952
C2 (moderate)	OC-SVM	[−2.66,−0.86]	[−3.96,−1.94]	[0.757,0.790]	[0.998,1.000]	[0.946,0.959]
			2/4 finite; 2 > +6 dB	0.544	0.807	0.747
C3 (adverse NLOS)	CAE	n.a.	[+5.60,+5.87]	[0.543,0.546]	[0.801,0.812]	[0.744,0.750]
			3/4 finite; 1 > +6 dB	0.542	0.732	0.717
C3 (adverse NLOS)	OC-SVM	n.a.	[+5.48,+5.98]	[0.535,0.549]	[0.706,0.758]	[0.702,0.732]

**Table 7:** Calibration transfer under four controlled single-parameter shifts. The detector models and p99 operating thresholds are obtained exclusively from the source-condition training and independent calibration partitions of each run and are transferred unchanged to the corresponding target condition. Values are means over five physically independent runs for both the CAE and OC-SVM, with brackets denoting 95% run-level Student-*t* confidence intervals (N=5, four degrees of freedom). All nominal source and target parameters are held fixed across runs, whereas packet, channel, noise, interference, synchronization, jammer, and model-initialization realizations are generated independently.

Controlled Shift	Target Condition	Detector	Clean Target FPR	$P_{D}\left(-10\right)$	$P_{D}\left(0\right)$	$P_{D}\left(+6\right)$	AUC (+6)
SNR	0→+8 dB	CAE	0.4617 [0.4094, 0.5140]	0.3646 [0.3186, 0.4106]	0.7999 [0.7527, 0.8471]	1.0000 [1.0000, 1.0000]	0.9844 [0.9628, 1.0000]
SNR	0→+8 dB	OC-SVM	0.4229 [0.3854, 0.4604]	0.3399 [0.3063, 0.3735]	0.7559 [0.7140, 0.7978]	0.9993 [0.9986, 1.0000]	0.9898 [0.9877, 0.9920]
CCI	−3→−9 dB	CAE	0.0127 [0.0047, 0.0207]	0.0105 [0.0029, 0.0181]	0.2852 [0.1865, 0.3839]	0.9938 [0.9887, 0.9989]	0.9997 [0.9995, 0.9999]
CCI	−3→−9 dB	OC-SVM	0.0130 [0.0047, 0.0213]	0.0125 [0.0050, 0.0200]	0.2527 [0.1827, 0.3227]	0.9794 [0.9719, 0.9869]	0.9984 [0.9978, 0.9989]
Nominal CFO	+1000→+500 Hz	CAE	0.0091 [0.0015, 0.0167]	0.0079 [0.0003, 0.0155]	0.2655 [0.1678, 0.3632]	0.9929 [0.9897, 0.9961]	0.9998 [0.9996, 0.9999]
Nominal CFO	+1000→+500 Hz	OC-SVM	0.0096 [0.0043, 0.0149]	0.0083 [0.0024, 0.0142]	0.2215 [0.1602, 0.2828]	0.9791 [0.9713, 0.9869]	0.9986 [0.9982, 0.9990]
Fading law	Rayleigh → Rician, K=6 dB	CAE	0.0138 [0.0021, 0.0255]	0.0108 [0.0014, 0.0202]	0.3155 [0.2177, 0.4133]	0.9950 [0.9918, 0.9982]	0.9997 [0.9995, 0.9999]
Fading law	Rayleigh → Rician, K=6 dB	OC-SVM	0.0130 [0.0040, 0.0220]	0.0117 [0.0042, 0.0192]	0.2717 [0.2122, 0.3312]	0.9835 [0.9766, 0.9904]	0.9986 [0.9980, 0.9992]

## Data Availability

The metric tables, per-condition detector scores and the scripts that regenerate every figure of this article are provided as Supplementary Material. Raw in-phase/quadrature captures are not redistributed because of their size but can be regenerated from the published generation parameters.

## Supplementary Materials

The following supporting information can be downloaded at: https://www.mdpi.com/article/10.3390/s26196123/s1, File S1: Source figures, numerical tables, documentation, checksums, and scripts corresponding to Figure 1, Figure 2, Figure 3, Figure 4, Figure 5, Figure 6, Figure 7, Figure 8 and Figure 9 and Table 1, Table 2, Table 3, Table 4, Table 5, Table 6 and Table 7; Data S1: Packet-level CAE and OC-SVM scores, thresholds, realization identifiers, and split manifests from the five independent evaluation runs; Data S2: Packet-level PMR and preamble-SER records for the 13 isolated conditions and five independent runs.

## Abbreviations

The following abbreviations are used in this manuscript:

AUC	Area under the receiver operating characteristic curve
AWGN	Additive white Gaussian noise
CAE	Convolutional autoencoder
CCI	Co-channel interference
CFO	Carrier frequency offset
CSS	Chirp spread spectrum
IoT	Internet of Things
LPWAN	Low-power wide-area network
NLOS	Non-line-of-sight
OC-SVM	One-class support vector machine
PMR	Peak-to-mean ratio (dechirp domain)
ROC	Receiver operating characteristic
SER	Symbol error rate
SF	Spreading factor
SNR	Signal-to-noise ratio
STO	Sampling time offset

## Appendix A. Estimation of the Sensitivity Floor

Let

$${\left\{\left(L_{i},{\hat{P_{D}}}_{i}\right)\right\}}_{i=1}^{17},\;L_{1}<L_{2}<\ldots <L_{17},$$

denote the measured detection characteristic over the relative jammer-level sweep. For a target detection probability δ, the sensitivity-floor estimator is defined from the first crossing of the prescribed criterion.

If ${\hat{P_{D}}}_{1}<\delta$ and the first crossing occurs between $L_{i-1}$ and $L_{i}$, the interpolated estimate is

(A1) $$\hat{L_{\delta }^{★}}=L_{i-1}+\frac{\delta -{\hat{P_{D}}}_{i-1}}{{\hat{P_{D}}}_{i}-{\hat{P_{D}}}_{i-1}}\left(L_{i}-L_{i-1}\right),\;i=\min\left\{i:{\hat{P_{D}}}_{i}\geq \delta \right\}.$$

The complete estimation and reporting rule, including the sweep boundaries, is therefore

(A2) $$\hat{L_{\delta }^{★}}=\left\{\begin{array}{ll} \leq L_{1},\; & {\hat{P_{D}}}_{1}\geq \delta ,\\ L_{i-1}+\frac{\left(\delta -{\hat{P_{D}}}_{i-1}\right)\left(L_{i}-L_{i-1}\right)}{{\hat{P_{D}}}_{i}-{\hat{P_{D}}}_{i-1}},\; & \begin{array}{l} i=\min\{i:{\hat{P_{D}}}_{i}\geq \delta \},\\ {\hat{P_{D}}}_{1}<\delta , \end{array}\\ \mathrm{not}\;\mathrm{attained},\; & {\hat{P_{D}}}_{i}<\delta \;\forall i. \end{array}\right.$$

The first case represents left censoring: if the target detection probability is already satisfied at $L_{1}=-10\;\mathrm{dB}$, the actual sensitivity floor may lie below the evaluated range and is therefore reported as

$$L_{\delta }^{★}\leq -10\;\mathrm{dB}.$$

Conversely, if the criterion is never reached by $L_{17}=+6\;\mathrm{dB}$, the floor is right-censored and reported as not attained.

The estimator uses the first crossing rather than the last whenever the empirical detection characteristic is non-monotone. This choice is relevant primarily in the very-low-SNR regime discussed in Section 4, where the directional behavior of PMR can invert. Retaining the first crossing is consistent with the early-warning interpretation because it identifies the earliest relative jammer level at which the prescribed detection requirement is satisfied.

The preamble-degradation onset $L_{0.05}^{\mathrm{SER}}$ is estimated using the same first-crossing interpolation procedure, replacing $\hat{P_{D}}\left(L\right)$ by

$$\Delta \hat{\mathrm{SER}}\left(L\right)=\hat{\mathrm{SER}}\left(L\right)-{\hat{\mathrm{SER}}}_{0}$$

and replacing the target δ by the selected degradation criterion of 0.05. This symmetric construction ensures that both quantities entering the early-warning margin,

$$M_{0.9}=L_{0.05}^{\mathrm{SER}}-L_{0.9}^{★},$$

are obtained from the same jammer-level grid using the same interpolation principle.

## Appendix B. Sensitivity of the Margin to the Degradation Criterion

The five-percentage-point increment adopted in Equation (7),

ΔSER=0.05,

is an operational convention used to identify a reproducible onset of jammer-induced preamble degradation; it is not intended as a universal LoRa link-failure threshold. To examine how this choice affects the resulting early-warning margins, the onset was also evaluated using the stricter one-percentage-point criterion

ΔSER=0.01.

The effect depends strongly on the nominal propagation condition. Under co-channel interference at CCI=0dB, the degradation onset shifts from

$$L_{0.05}^{\mathrm{SER}}=-1.87\;\mathrm{dB}$$

for the 0.05 criterion to

$$L_{0.01}^{\mathrm{SER}}=-2.82\;\mathrm{dB}$$

for the 0.01 criterion, a displacement of 0.94dB. The principal comparison with the CAE is unchanged. The CAE does not reach the detection criterion under either definition, so its margin remains not attained. By contrast, the PMR margin decreases from +6.58dB to +5.64dB, and the OC-SVM, which remains left-censored at $L_{0.9}^{★}\leq -10\;\mathrm{dB}$, retains an early-warning margin of at least 7.18dB under the stricter degradation definition. Thus, the large sensitivity separation between the CAE and the two more sensitive detectors under strong co-channel interference is not an artifact of the five-percentage-point criterion.

The dependence is substantially stronger under severe Rayleigh fading. There, the onset moves from

−1.75dB

to

−3.33dB

when the criterion is tightened from 0.05 to 0.01, a shift of 1.58dB. This behavior reflects the fact that the nominal preamble is already degraded before jamming is introduced, with ${\mathrm{SER}}_{0}=0.124$, so a small additional error increment is reached rapidly. Under the stricter criterion, the CAE margin decreases from −0.22dB to approximately −1.80dB, whereas the OC-SVM margin decreases from −7.58dB to approximately −9.16dB.

The sensitivity analysis therefore leads to two conclusions. First, the absolute value of the early-warning margin necessarily depends on the selected communication-side degradation criterion, particularly in channels whose nominal preamble is already impaired. Second, the principal comparative conclusions remain intact: PMR and the OC-SVM retain a large advantage over the CAE under strong co-channel interference, while severe multipath sharply compresses the available warning region and continues to favor the CAE over the OC-SVM and PMR. The margin should therefore be interpreted together with its stated degradation criterion rather than as an absolute detector property independent of the protected communication condition.

Per-condition degradation onsets under both the 0.05 and 0.01 criteria are provided in the supplementary metric tables.
